# Comprehensive geriatric assessment and management in primary care: a systematic literature review with a descriptive mapping of team composition and assessment instruments

**DOI:** 10.3389/fpubh.2026.1739380

**Published:** 2026-03-20

**Authors:** Francesca Fulceri, Angela Caruso, Martina Micai, Maria Vittoria Notari, Camilla Cocchi, Ambrogio Cerri, Graziano Onder, Maria Luisa Scattoni

**Affiliations:** 1 Coordination and Promotion of Research, Istituto Superiore di Sanità, Rome, Italy; 2 Università Cattolica del Sacro Cuore, Rome, Italy; 3 Department of Biomedicine and Prevention, Doctoral School of Nursing Sciences and Public Health, University of Rome Tor Vergata, Rome, Italy; 4 Fondazione Policlinico Universitario A. Gemelli IRCCS, Rome, Italy; 5 Istituto Superiore di Sanità, Viale Regina Elena, Rome, Italy

**Keywords:** care plan, comprehensive geriatric assessment, frailty, older people, systematic review

## Abstract

**Introduction:**

Comprehensive Geriatric Assessment and Management (CGAM) is a multidimensional, interdisciplinary diagnostic process to create a coordinated care plan for vulnerable older adults by addressing their medical, psychological, and functional needs. This systematic review examines randomized trials comparing CGAM with standard care or alternative interventions for older individuals in primary care focusing on how CGAM is operationalized through professional team composition and assessment instruments rather than on effectiveness outcomes alone.

**Methods:**

A search across databases identified 3,112 studies, leading to 31 studies being included in the literature review. Eligible studies generally included participants aged 65 years and older. Across all included studies the mean age of participants was above 70 years.

**Results:**

The findings show a considerable variation in team composition and assessment instruments. While this heterogeneity reflects contextual adaptability, it also highlights the lack of shared standards for CGAM implementation in primary care. Most teams consisted of three to five members. The most frequently involved professionals were nurses/licensed practical nurses (*n* = 27 studies), geriatricians (*n* = 21), and social workers (*n* = 14). General practitioners were involved in more than half of the included studies. Different assessment instruments employed in the studies addressed multiple dimensions of older adults’ health, including physical, cognitive, emotional, and social functions.

**Discussion:**

The variability in team composition and assessment instruments highlights the adaptability of CGAM across healthcare settings, emphasizing the need to balance standardization with the flexibility required to meet users’ needs and available resources. Based on the available evidence, future CGAM research and practice should prioritize the definition of core team components, the harmonization of assessment domains and instruments, and the development of implementation-ready models that balance standardization with local flexibility.

## 1 Introduction

Normal aging is a multidimensional process, whereas frailty represents a pathological deviation marked by reduced physiological reserve and increased vulnerability, commonly conceptualized through the physical phenotype and the deficit-accumulation index (1, 2). Recent international reports underscore frailty as a major global geriatric syndrome with substantial clinical, social, and policy implications, highlighting the need for early detection, multidimensional assessment, and coordinated interdisciplinary care in primary care settings (3). Comprehensive Geriatric Assessment and Management (CGAM) is a multidimensional, interdisciplinary diagnostic process designed to develop an integrated care plan for frail older individuals, considering their medical, psychological, and functional status (4, 5). CGAM evaluates frail individuals and serves as a framework to develop personalized interventions that address their specific needs. Evidence indicates that CGAM enhances health outcomes in frail, community-dwelling older adults at risk of adverse health events (4). As life expectancy increases, there is a growing need to identify effective strategies for delivering appropriate healthcare that meets the needs of older individuals at risk of poor health outcomes and loss of independence. Much of the research in this field primarily focuses on the effectiveness of CGAM, as assessed by different clinical outcomes including hospital admissions, medical interventions, and mortality rates (4, 6, 7). However, the optimal team composition and the most suitable assessment instruments for integrated care plans implementation and monitoring remain undefined. The heterogeneity observed across CGAM models is consistent with the longstanding lack of consensus on frailty definitions, as highlighted by systematic reviews showing substantial conceptual variation in how frailty is operationalized in clinical practice (8). Moreover, the heterogeneous manifestations of frailty across regions and populations call for research that better reflects differences in geography, health systems, community settings, and policy priorities (9).

In Italy, the Italian National Health Service (INHS), under Article 27 of Decree No. 29 (March 15, 2024), which implements Law No. 33 (March 23, 2023), has introduced policies to ensure equitable access to tailored social and healthcare services for older adults’ needs. Within this framework, the Italian National Institute of Health (Istituto Superiore di Sanità, ISS) is tasked with establishing evidence-based criteria to define the healthcare professionals and assessment instruments required for CGAM to develop individualized care plans. The Italian National Guidelines emphasize the importance of its implementation across various healthcare settings, including outpatient and primary care/general practice (7). These recommendations, derived from randomized controlled trials (RCTs) comparing Comprehensive Geriatric Assessment to standard care and observational studies, are based on the accuracy and predictive value of multidimensional assessments for adverse health outcomes. Developed using the Grading of Recommendations Assessment, Development, and Evaluation (GRADE) methodology, the guidelines were created by an expert panel, which includes representatives from leading Italian scientific and professional healthcare organizations, General Practitioners, Primary Care providers, and Geriatrics Hospital-Community Societies, in collaboration with ISS experts in the field and in the methodological application of research to public health services.

This work contributes to the existing literature by providing a comprehensive overview of CGAM, emphasizing its role in facilitating the development of integrated, individualized care plans that enhance the health and well-being of older adults. Building on the work of Briggs et al. (4), this systematic literature review aims to update the literature by exploring more recent studies on individual and clustered randomized trials that compared CGAM to usual care or other interventions for older adults in primary care. While previous systematic reviews, have primarily focused on the effectiveness of CGAM in reducing adverse clinical outcomes, considerably less attention has been devoted to understanding how CGAM is operationalized in primary care settings. In particular, uncertainty remains regarding which healthcare professionals should be involved and which assessment instruments are most frequently used to inform individualized care plans. These gaps represent a barrier to the translation of evidence into standardized practice and policy, especially in primary care systems facing workforce and resource constraints.

Therefore, the present literature review aims to move beyond efficacy outcomes and to provide a systematic characterization of the structural components of CGAM in primary care, focusing on team composition and assessment instruments as key determinants of implementation.

## 2 Methods

The present systematic literature review followed the Preferred Reporting Items for Systematic Reviews and Meta-Analyses (PRISMA) statement and was designed to provide a descriptive and systematic characterization of CGAM team composition and assessment instruments, rather than a quantitative synthesis of intervention effectiveness. The systematic literature review was registered in the International Prospective Register of Systematic Reviews (PROSPERO) database (registration number: CRD42024562519). The systematic review team at ISS included expert researchers, clinicians, and methodologists. Table 1 reports the key elements of the review protocol. The ISS review team developed the search strategy based on the framework established by the Cochrane Review (4). The strategy was further refined in collaboration with ISS expert librarians to incorporate natural language and Medical Subject Headings (MeSH) terms. Details on search strategies are reported in Supplementary material.

**Table 1:** Key elements of the review protocol.

PICO component	Description
Participants	Participants aged 65 years or older (or 55 years or older if the mean age of study participants was over 70 years); Community dwelling; Not acutely unwell (i.e., not currently an inpatient in an acute hospital and not presenting to an emergency department or general practitioner for unscheduled care); Identified as at risk of nursing home admission or defined as frail.
Intervention	Comprehensive Geriatric Assessment used to inform a holistic care plan.
Comparator	Usual care or other interventions.
Context	Either the participant’s own home or other community settings. Research in low- and middle-income countries were included.
Outcome	Team composition and assessment instruments.
Study design	Individual and clustered randomized trials that compared intervention to usual care or other interventions.

From April 1, 2020, to January 22, 2026, a comprehensive literature search was conducted across multiple databases, including the Cochrane Library, MEDLINE, Embase (via Ovid), CINAHL (EBSCOhost). Additional sources were searched, including ClinicalTrials.gov, the International Clinical Trials Registry Platform (ICTRP),^1^ and the McMaster Aging Portal.^2^ No language restrictions were applied. All identified records were imported into the Rayyan QCRI systematic review web application (10), where a researcher removed duplicates.

Two or more independent reviewers from the review team (AC, AG, CC, FF, MM, MV) screened each title and abstract based on the inclusion criteria (Table 1). In line with Briggs et al. (4), we excluded the following types of studies: studies that focused solely on a single disease or syndrome (e.g., heart failure, falls, stroke); studies of interventions after discharge from hospital; studies designed to test hospital avoidance in exacerbations of chronic conditions; studies involving participants who were not community-dwelling. Two independent authors reviewed the selected records in full text to identify the studies to be included. The reference lists of the review studies identified through the search were manually reviewed to identify additional relevant literature.

Two authors independently extracted the characteristics and outcome data from each included study. They also cross-checked and extracted any relevant information or outcomes from the studies included by Briggs et al. (4). Discrepancies during the screening, selection, or data extraction process were solved through discussion, when necessary, by consulting a third author.

The risk of bias in the included RCTs was assessed by two independent researchers using the Jadad’s, which represents the estimation of the robustness of a clinical trial by a numerical value (11, 12). The Jadad score ranges from 0 to 5 and is based on five criteria, with points assigned based on their presence or absence. A higher score indicates a well-designed clinical trial characterized by randomization, double-blinding, a clear explanation of the randomization and blinding methods, and a detailed account of study withdrawals.

Given the heterogeneity of interventions and the descriptive focus of this review, results are presented as a narrative and tabular synthesis rather than as a meta-analysis of effect sizes.

## 3 Results

**Figure 1:**
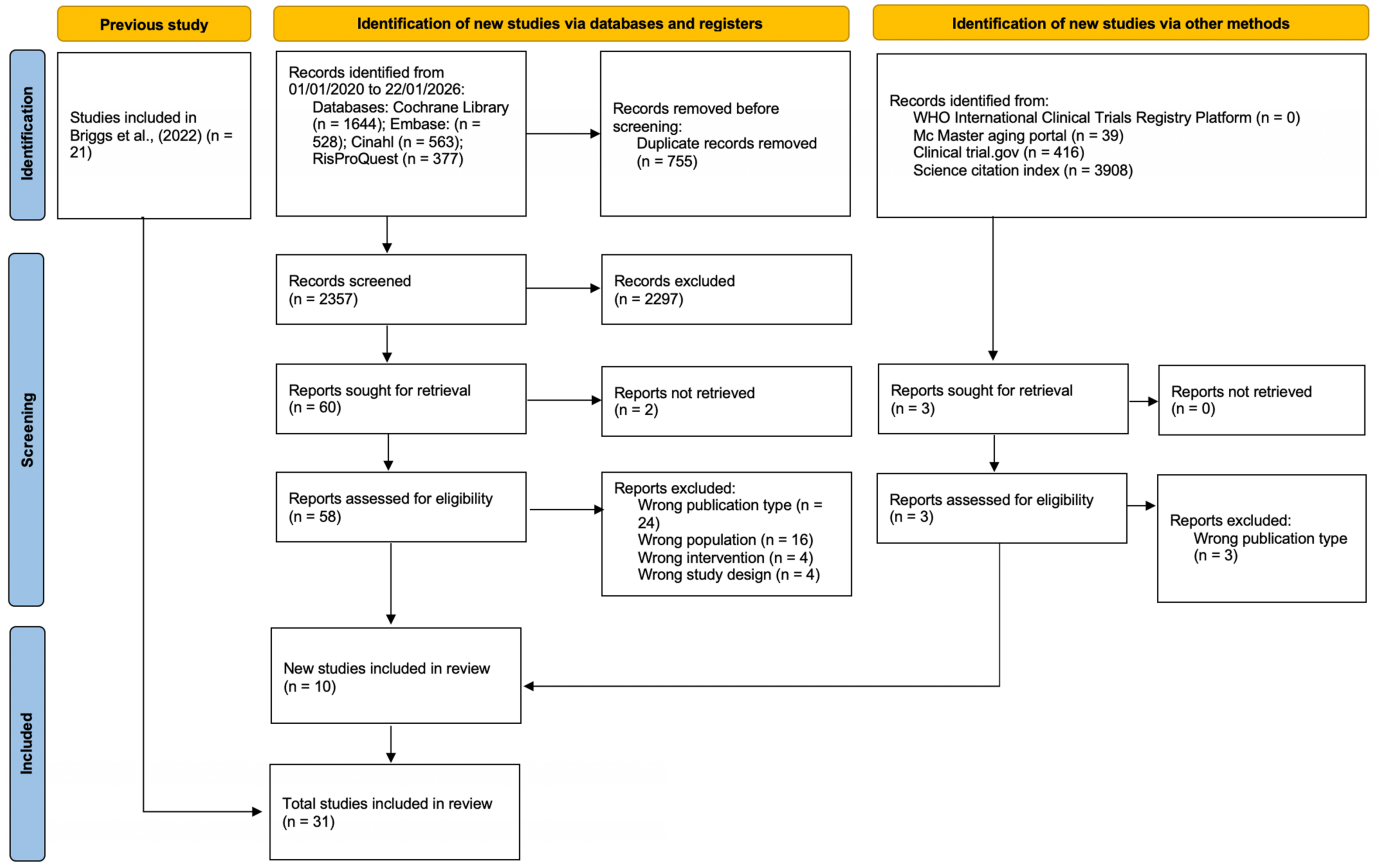
PRISMA 2020 flow chart diagram for updated systematic reviews which included searches of databases, registers, and other sources.

**Figure 2:**
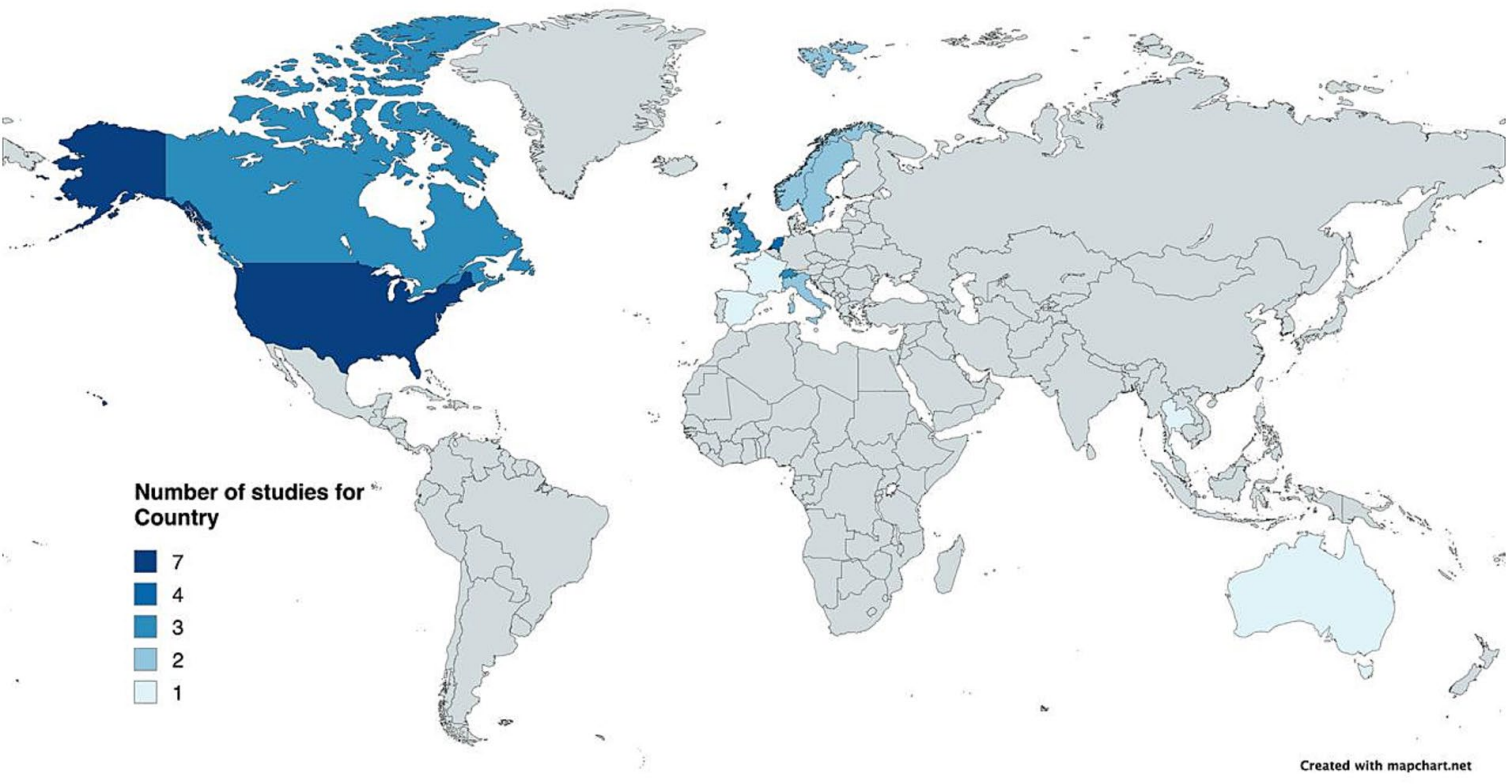
Geographic locations of the included studies.

The database search identified 3,112 studies, with 755 duplicates removed. Two independent reviewers screened the titles and abstracts of the remaining 2,357 studies based on predefined inclusion criteria, excluding 2,297 studies. Despite contacting the authors, the full text of two studies was not retrieved. The remaining 58 studies underwent full-text assessment by two independent reviewers. Finally, 31 studies were included, and two independent reviewers extracted their data. The majority of the studies were conducted in Europe (*n* = 18), followed by North America (*n* = 11), and a limited representation from Australia (*n* = 1) and Asia (*n* = 1). This distribution indicates a strong concentration of evidence from high-income Western healthcare systems. Figure 1 presents the flowchart following the PRISMA 2020 flow diagram for systematic literature reviews detailing each stage’s study screening and selection process. Figure 2 reports the geographic locations of the studies included. Table 2 summarizes the study’s characteristics of the included studies. Eligible studies included participants aged 65 years and older, with the exception of two studies that applied broader criteria, allowing the inclusion of individuals aged 55–65 years if they met additional requirements and demonstrated care needs. Across all included studies the mean participant age was above 70 years (see details in Supplementary Table 1). The detailed reporting of inclusion and exclusion criteria of the included studies is reported in Supplementary Table 2. The methodological assessment of the quality of the studies is reported in see Supplementary Table 3.

**Table 2:** Study’s characteristics: intervention applied to the experimental group and the usual standard care provided to the control group.

Study		Experimental group		Control group
	n°	Description of CGAM intervention	n°	Description of usual care or other interventions, if available
Bernabei et al. (37)	99	Case management and care planning by the community geriatric evaluation unit and GP	100	PC and community care—conventional services’ organization; GP’s ambulatory/HV, nursing, social services, home aids, meals on wheels
De Luca et al. (13)	30	Follow-up (1 yr) by different professionals. e-Service: neurological consultation, nutritional assessment, psycho-social interventions. Interviews on daily difficulties and telemedicine management; additional contact by staff. Telecounselling 3/ wk. (up to 45′ videoconference). Monitoring and counseling by nurses (2–3/wk)	30	Territorial care (2-3/mo for 1 yr). Outpatient service commonly provided (not always accessible for personal/geographical barriers). Neurological/geriatric visit, psychosocial interventions, besides the basic care/cure
Boult et al. (20)	294	Outpatient geriatric evaluation and management	274	Usual health care
Brazil et al. (21)	32	Nurse obtained a medical summary from the GP, organized HV for assess and design a person-centered care plan (emphasis on personalized care style, facilitate the dialogue with the patient/family carer about current and future care needs/goals). The patient’s medication list was review based on guidelines. The nurse drafted a report and informed GP who recommended actions, provided feedback, and confirmed the care plan	31	Usual care (request of appointments with the GP to discuss any health problems)
Clarkson et al. (31)	129	Integrated assessment: care managers’ assessments with additional specialist clinical assessment by an old age psychiatrist or geriatrician. Note: integration of care management (old age psychiatry, geriatric medicine and social services)	127	Usual care management assessment by care managers to beat risk of care-home entry
Counsell et al. (22)	474	Geriatric Resources for Assessment and Care of Elders (GRACE) model (Home-based care intervention)	477	Usual care
Di Pollina et al. (40)	122	HV by a multidisciplinary geriatric team with access to a call service and coordinated follow-up; 2 HV by nursing service and CGU that included a physician to perform in-home multidimensional GA, and a 24 h/7 day a week call service	179	Usual care by the PCP and home visiting nursing services
Ekdahl et al. (39)	208	CGAM-based care (24 to 31 mo) at the geriatric ambulatory unit in addition to usual care	174	Usual care
Engelhardt et al. (14)	80	CGAM as outpatient with care plan and periodic reassessment	80	Usual PC
Fairhall et al. (38)	120	Interdisciplinary multifactorial intervention incorporating principles of geriatric evaluation and management	121	Usual care from community services and GP (assessment and delivery of care needs; medical health management)
Federman et al. (27)	114	HBPC involves multidisciplinary teams who provide care, and the intensive care coordination required for ill adults at home (out of long-term care facilities)	112	Usual care with the usual provider
Fristedt et al. (23)	31	HV (geriatrician and nurse), CGA administered and individualized care plan formulated. Note: MGT included physicians and nurses, occupational therapists and physiotherapists at times (Swedish health and social care authorities), conduct CGAM at home and (based on the results) develop and implement sustainable/coordinated care plans	31	Standard care based on the individual needs from the hospital or PC, including healthcare or social care provided by the municipality or private companies
Hoogendijk et al. (24)	1,147	Multidimensional GA by practice nurse, informing tailored care plan, linked with expert geriatric team; tailored care plan created with PCP. Note: The GCM combines tailored care based on GA with management by geriatric expert teams. GCM was implemented in PC for community-dwelling frail older adults		Usual care
Imhof et al. (32)	231	In-home nurse-delivered Health Consultation Program; family physicians and community nurses provided basic healthcare services. Note: HCP comprises a standardized CGAM, evidence-based guidelines for health problems, 4 home consultation, and 3 follow-up phone calls over 9 mo	230	Healthcare services as usual provided by community health nurses and physicians, and covered by the participants’ mandatory health insurance
Li et al. (15)	152	CGAM with appropriate intervention when indicated based on assessment results. Note: CGAM and intervention in pre-frail and frail community-dwelling older adults (Fried Frailty Criteria and Barthel Index)	158	Screening evaluation only
Lyndon et al. (36)	30	Program of HV by Community Matrons possessing advanced assessment and non-medical prescribing skills; person centered intervention consisting of a holistic assessment based on a conversation (participant and nurse) for a personalized plan of care developed with the participant and referral to other services as required. Intervention dose max: 1 assessment and 6 care planning visits conducted over a 12 wk.	26	Standard PC for frailty. Approaches to care is variable PC (British Geriatrics Society et al., 2014) and may include the management of long-term conditions, referrals to other services, prescribing of medications and routine vaccinations delivered by a GP or other PC clinician.
Mangin et al. (29)	257	Health Teams Advancing Patient Experience: Strengthening Quality (Health TAP ESTRY) a complex PC program aimed at assisting older adults to stay healthier for longer	255	Usual care
Melis et al. (16)	85	Multidimensional assessment by geriatric nurse specialist in home, informing individualized treatment plan; PCP continued to be responsible for the patient care. Note: The Dutch Geriatric Intervention Program is a multidisciplinary community intervention model, consisting of nurse HV.	66	Usual care
Metzelthin et al. (43)	193	CGA by practice nurse in patient’s home, used to formulate treatment plan; the GP and practice nurse built the core team of the interdisciplinary care approach Note: Prevention of Care” (PoC) approach focuses on both older people with an increased risk for developing disability and older people who are already disabled	153	Usual care
Monteserin et al. (33)	308	CGAM followed by individual sessions with geriatrician	312	Usual standard care from GP
Montgomery and Fallis (17)	82	Multidimensional assessment by a trained coordinator who enhanced access to geriatric/DH services; care plan developed by the coordinator and reviewed by geriatrician/DH team. Home assessment by the geriatrician/team member; DH assessment by team members; referral to appropriate home and community-based service	82	Assessment and follow-up by home care coordinator in the usual
Mueller et al. (34)	217	Yearly assessment by GP of 8 geriatric syndromes and associated tailored management plan. The instrument can be integrated in GP practices without the need for additional organizational changes. AGE instrument, specifically designed for GPs consists of a brief assessment of the most relevant geriatric syndromes combined with management plans	212	Routine care
Orcel et al. (30)	421	Nurse-led CGA (n°231): systematic CGA performed by a trained nurse following a 1-day seminar; assessment completed within 1 mo and used to develop a personalized care plan; geriatric hotline available for GP support. GP-led CGA (n°190): CGA performed by the GP on a case-by-case basis after the same 1-day seminar; personalized care plan developed directly by the GP; geriatric hotline accessible throughout follow-up.	213	Usual care
Reuben et al. (35)	180	CGAM consultation and intervention to achieve adherence to recommendations from the CGAM; the geriatrician leading the assessment telephoned the subject’s PCP to convey the CGAM recommendations. The personal approach to CGAM consultation allowed the PCP to provide input regarding the appropriateness of CGAM recommendations	183	In-depth, standardized, CGAM from social worker, gerontologic nurse, practitioned geriatrician team, physical therapist at community-based clinic. A short interdisciplinary case conference followed evaluations
Rockwood et al. (41)	95	Mobile geriatric assessment team delivering CGAM	87	Usual care
Romskaug et al. (28)	87	GA: medical history, systematic screening for problems, clinical examination, supplementary tests, medication review (emphasis on indication, dosage, adverse effects, interactions). Meeting between geriatrician and FP for the plan. Follow-up by the FP	87	Usual care
Safari et al. (42)	35	2 ANPs specializing in the health care of older adults assess participants at homes/GP centers; CGAM (assessment, matters important to participants, care and support plan protocol). ANPs produced a personalized goal-oriented care and support plan incorporating a self-care program. If needed ANPs referred to other specialists (as a hub)	37	Treatment as usual
Silverman et al. (18)	239	Outpatient GA based on a consultative model conducted at 1 of 4 geriatric assessment units. Team provided evaluation (medical, psychological, social health problems) and comprehensive treatment plan. The assessment was concluded with a family conference	203	Usual care from physicians in the community
Sommers et al. (19)	280	Care from their PCP working with registered nurse and social worker; this office-based intervention demanded close collaboration among PCP. Note: interdisciplinary, collaborative, practice intervention involving PCP, nurse, social worker	263	Care as usual from their PCP
Spoorenberg et al. (25)	747	Multidisciplinary Care Team–consisting of the older adults’ GP, a nursing home physician and two case managers (district nurse and social worker)–provides care and support to older adults	709	Care provided by GP and local health and community organizations. Once a health problem is found, patients enter the health care system–in most cases with a visit to their GP
Stensvik et al. (26)	159	Modified Comprehensive Geriatric Assessment using validated instruments (physical and psychological health problems) followed by structured Case Conferencing-meeting to discuss and develop an individual care plan	150	Usual care

CGAM, Comprehensive Geriatric Assessment and Management; CGU, Community Geriatric Unit; GA, Geriatric Assessment; GEM, Geriatric Evaluation and Management; GP, General Practitioner; HV, Home Visit; mo, Months; PC, Primary Care; PCP, Primary Care Physician; wk, Weeks; yr, Years.

### 3.1 Team members and professional composition

The composition of the CGAM healthcare team for each study is reported in Table 3. Collected data are based on information provided in the methodology sections of the studies. When the number of professionals was not explicitly stated, it was assumed that each mentioned profession represented one unit. Thus, the CGAM composition estimates should be interpreted as reflecting the minimum number of professionals included in the healthcare team for each study. The involvement of General Practitioners is also reported, and where applicable, their specific role within the team is detailed.

**Table 3:** CGAM team and GP involvement.

Study, country	Healthcare equipe	General practitioner involvement
	Expertise (n°)*	Role in care plan definition and/or monitoring
Bernabei et al. (37), Italy	Case Managers (2); GPs; Geriatrician (1); Nurses (1); SW (1)	Described as a component of the team. Involved in the care plan; Case Managers relied on GP evaluation
Boult et al. (20), USA	Geriatrician (1); Gerontological Nurse Practitioner (1); Nurse (1); SW (1)	No
Brazil et al. (21), Ireland	Nurse—trained study (1); Pharmacist (1); GP (1); GP Practice Manager (1)	Described as a component of the team. Received assessment results, recommended actions, provided feedback, confirmed the care plan
Clarkson et al. (31), UK	Care Manager (1); Geriatrician/Old Age Psychiatrist (>1)	GP received a copy of assessment
Counsell et al. (22), USA	Geriatrician (1); Nurse-advanced practice (1); Primary Care Physician (1); SW (1)	Described as a component of the team. Initial visit before recruitment and collaboration with the support team
De Luca et al. (13), Italy	Psychologist (1); Nutritional Biologist (1); SWs; Psychiatrists^§^; Nurses	No
Di Pollina et al. (40), Switzerland	Intervention Nursing Team (1); Primary Care Physician (1); Community Geriatrics Unit: Dieticians, Doctors, Occupational and Physical Therapists, Psychologists, SWs	Described as a component of the team. Followed patients with the community geriatric unit
Ekdahl et al. (39), Sweden	Ambulatory Geriatric Unit (Ekdahl, 2015^): Nurse, Geriatrician/Resident Physician, Municipal Care Manager, Occupational, Therapist, Physiotherapist, Dietician, Administrative Assistant	No
Engelhardt et al. (14), USA	Board-Certified Geriatrician (1); Nurse -Practitioner (1); SW (1)	No
Fairhall et al. (38), Australia	Dietician (1); Geriatrician (1); Nurse (1); Physiotherapists (2); Rehabilitation Physician (1)	No
Federman et al. (27), USA	Physician (1); Nurse Practitioner (1); Nurse (1); SW (1); Administrative assistant (1)	No
Fristedt et al. (23), Sweden	Nurse (1); Occupational Therapists; Physicians or Geriatrician (1); Physiotherapists	No
Hoogendijk et al. (24), The Netherlands	Nurses-trained practice; Primary Care Physicians; Nurse-Geriatric (1); Geriatrician (1)	Described as a component of the team; worked with nurses based at the primary care practices; carried out the intervention; reviews the outcomes of the assessment
Imhof et al. (32), Switzerland	Nurse -Gerontological (1); Doctor specialized in Geriatrics (1)	No
Li et al. (15), Taiwan	Geriatricians (2); Nurses	No
Lyndon, 2023, UK	Advanced practitioner nurses with advanced diagnostic and prescribing skills known as Community Matrons	Data entering in a customized database
Melis et al. (16), The Netherlands	Nurse -Geriatric specialist (1); Geriatrician (1); Primary Care Physician (1)	Described as a component of the team. Enrollment, initiate the intervention, made referrals, medication changes, interdisciplinary consultations; responsible for the care and final decisions
Mangin et al. (29), Canada	At least three different health care team members: Administrative assistant, Chemical dependency counselor, Chiropody, Community outreach nurse, Registered dietician, Health Promoter, Kinesiologist, Mental Health Nurse, Nurse practitioner, Occupational Therapist, Pharmacist, Physician, Physician assistant, Physiotherapist, Registered Nurse, Registered practical nurse, Respiratory Therapist, SW, System Navigator, Volunteer Coordinators	Not specified
Metzelthin et al. (43), The Netherlands	GP (1); Nurse -Practice (1); Physical Therapists; Occupational Therapists; Geriatrician^§^ (1); Pharmacist^§^ (1)	Described as a component of the team with practice nurse; GP cooperates with occupational and physical therapists
Monteserin et al. (33), Spain	Geriatrician (1); Nurse (1)	Medical record with specific recommendations for evaluation and management of interest to the patient’s GP
Montgomery and Fallis (17), Canada	Coordinator (1); Geriatrician (1); Day-Hospital Team (1)	No
Mueller et al. (34), Switzerland	GPs; Medical Assistants	Described as a component of the team. Recorded adverse events; administered (could delegate to medical assistants)
Orcel et al. (30), France	Nurse (1); GP (1); Geriatricians	Described as a component of the team in the GP-led CGA. Described as responsible for review and validation in the nurse-led CGA.
Reuben et al. (35), USA	SW (1); Gerontological Nurse Practitioner/Geriatrician Team (1); Physiotherapist^§^ (1)	Implementation of CGAM recommendations
Rockwood et al. (41), Canada	Nurse Geriatric assessors (2); Geriatricians (4); Physiotherapist (1); Occupational Therapist (1); SW (1); Dietitian (1); Audiologist (1); Speech Language Pathologist (1)	Recruitment. Involvement in case of urgent or critical situation (requiring immediate assessment or hospitalization)
Romskaug et al. (28), Norway	Physician trained in Geriatric Medicine (1); Senior Consultant (1); GP (1)	Described as a component of the team. Performed a supervised assessment; meeting between the geriatrician and follow up
Safari et al. (42), UK	Nurse—advance Nursing practitioners; Geriatrician^§^ (1); Pharmacist^§^ (1); Physiotherapist^§^ (1); Psychiatrist^§^ (1); Occupational Therapist^§^ (1); SW^§^ (1)	Involved in the process of identifying eligible subjects
Silverman et al. (18), USA	Internist with a specialty in geriatric medicine (1); Nurse Geriatric (1); SW Geriatric (1)	No
Sommers et al. (19), USA	GP (1); Nurse Geriatric (1); SW	Described as a component of the team
Spoorenberg et al. (25), The Netherlands	Older Adult’s GP (1); Nursing Home Physician (1); District Nurse (1)—Case Manager Role; SW (1)—Case Manager Role	Multidisciplinary care team member
Stensvik et al. (26), Norway	Nurse Registered (1); Licensed practical Nurses (2); Assistants	No

GP, general practitioner; SW, social worker; ^§^if needed. *The number in parentheses indicates the number of professionals, when stated in the article. When the number of professionals was not explicitly stated, it was assumed that each mentioned profession represented one unit. For instance, if a study mentioned “nurses” without specifying the number, it was counted as “one nurse.” Thus, the CGAM composition estimates should be interpreted as reflecting the minimum number of professionals included in the healthcare team for each study (39).

The studies (*n* = 18) reported that the number of professionals participating in the GCAM intervention ranges from three to five (13–30). Furthermore, six studies indicated fewer than three professionals (31–36) in the team, while five described teams with more than five members (37–41). One study specified a range from one to six professionals, and another reported a range of 4–6 (42, 43) professionals in the team.

**Figure 3:**
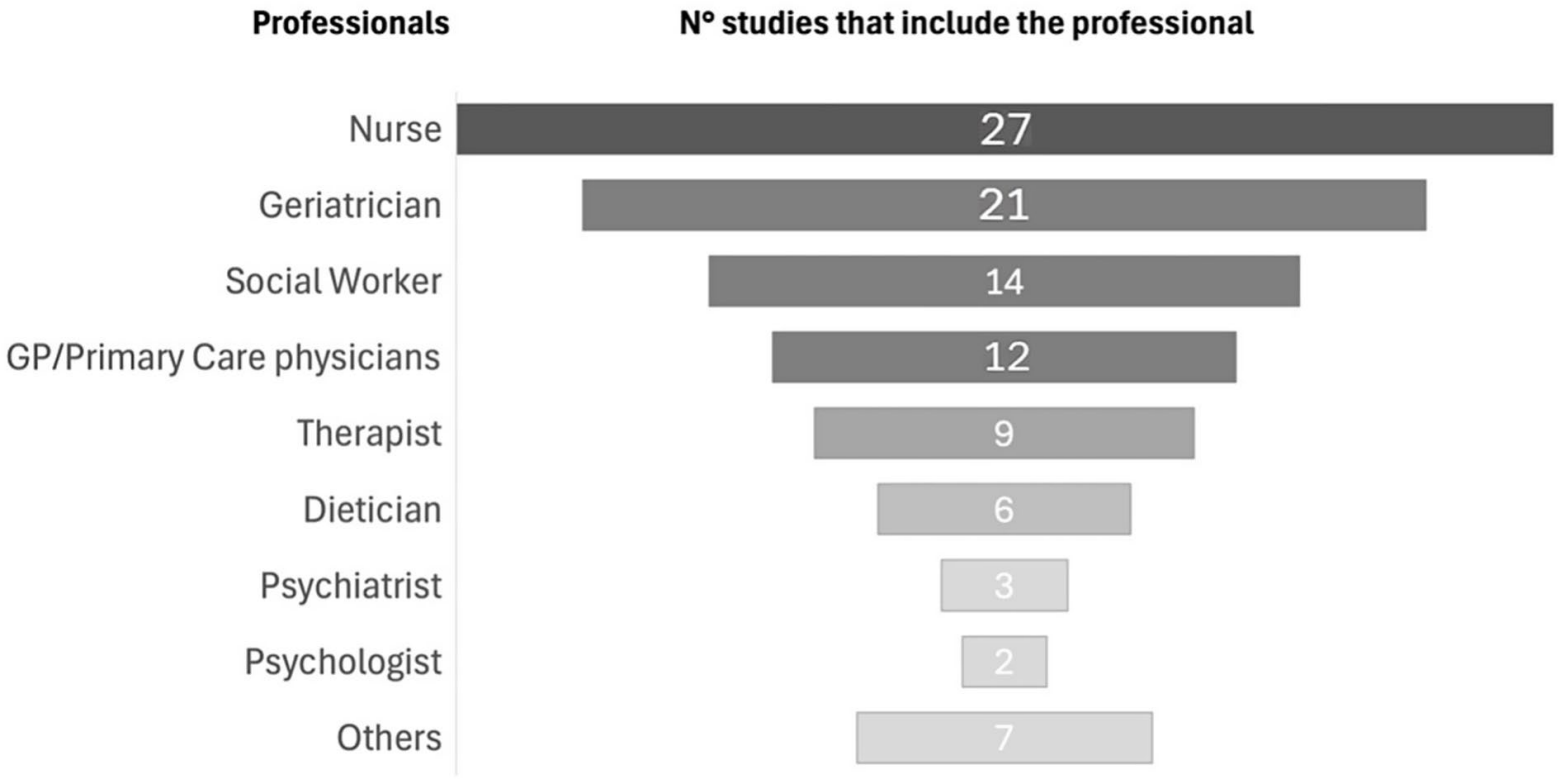
CGAM professional composition team.

The most frequently involved professionals were nurses/licensed practical nurses (*n* = 27 studies), geriatricians (*n* = 21), and social workers (*n* = 14). Other healthcare team members, including therapists (*n* = 9), dietitians/nutritional biologists (*n* = 6), pharmacists (*n* = 4), psychiatrists (*n* = 3), and psychologists (*n* = 2) were part of the team. Some studies mentioned additional roles, such as neurologists, case managers, and administrative staff. Figure 3 shows the number of studies that include the professionals.

Across studies, geographic variation emerged in the organizational embedding of CGAM teams. European studies tended to report more heterogeneous, multidisciplinary team compositions, frequently including nurses, geriatricians, General Practitioners, and allied health professionals such as occupational and physical therapists. In contrast, North American studies more commonly reported compact teams, typically composed of a physician or geriatrician, a nurse or nurse practitioner, and a social worker.

### 3.2 Comprehensive geriatric assessment instruments

Table 4 provides a detailed overview of the assessment instruments used to implement the CGAM-based intervention plan for older adults. Collected data are based on information provided in the methodology sections of the studies. The used instruments vary across studies and cover different assessment domains, including physical function and activities of daily living (e.g., Barthel Index, Index of ADL, Timed Up and Go test), cognitive function and emotional state (e.g., Mini-Mental State Examination—MMSE, Short Portable Mental Status Questionnaire—SPMSQ, Geriatric Depression Scale—GDS), nutritional status (e.g., Mini Nutritional Assessment—MNA), pain and medical symptoms (e.g., Visual Analogue Scale, Memorial Symptom Assessment Scale—MSAS), Social network and support (e.g., Lubben Social Network Scale—LSNS, Satisfaction with Support Scale—SSS), Medication use and therapy management (e.g., Medication review, CAGE questionnaire for alcohol consumption), and Quality of life and general well-being (e.g., EQ-5D, Spitzer Quality of Life Index, Duke Health Profile).

**Table 4:** CGAM assessment instruments by domain across included studies.

Study (year)	Cognition /mental health/mood	Functional status/physical/mobility	Social domain/support	Nutrition	Medication/frailty/other
Bernabei et al. (37)	SPMSQ GDS	ADL, IASDL	–	–	Diagnoses, drug treatments, GP home visits
Boult et al. (20)	MMS	ADL TUG, gait, and balance	LSNS, social network	Nutrition screening	Medications, CAGE, environment
Clarkson et al. (31)	MMSE, GDS	BI	LSNS	–	CAPEBRS, need shortfall rating
Counsell et al. (22)	Mental status, Affect	Functional assessment, gait, and balance,	Social supports	–	Medication review, orthostatic vital signs, vision, hearing, see Counsell et al. (22)
De Luca et al. (13)	MMSE GDS, BPRS	ADL, IADL	CBI	MNA	BANSS, SUS
Di Pollina et al. (40)	MMSE, Clock drawing GDS	ADL, IADL, TUG, Semi-tandem stand	–	MNA-SF	Pain (VAS), medication review
Ekdahl et al. (39)	MMSE, GDS	BI, Grip strength, walking speed	Social support, transportation, family/caregivers	BMI	Frailty (CHSA), EQ-5D Feeling of security and of quality of life, see Lind-Mazya et al. (56)
Engelhardt et al. (14)	GDS, BSI, PGCMSR	FIM	LSNS, SSS	–	Medical Outcomes Study SF, Health Survey, QAR, COC, PPI, SSQ, Patient Satisfaction Questionnaire
Fairhall et al. (38)	MMSE	Activities of daily living, Falls/fractures history	–	–	Fairhall et al. (38)
Federman et al. (27)	–	ADL, Fall risk	–	–	MOLST, Medical history, Physical exam
Fristedt et al. (23)	MMSE	ADL			See Ellis et al. (57)
Hoogendijk et al. (24)	interRAI CHA	interRAI CHA	interRAI CHA	interRAI CHA	interRAI CHA
Imhof et al. (32)	Cognition, GDS	TUG, tandem stand, mobility/falls, gait, balance and strength, timed five-chair-rise test, aides for mobility	Living situation, Family network	MNA	Vision (Amsler), substance use, pain, hearing ability, sleep pattern, bladder control, use of medications
Li et al. (15)	MMSE GDS	TUG, FRT, falls	–	MNA	Orthostatic hypotension, visual acuity test, incontinence, polypharmacy, sleep disturbance, and pain conditions
Lindon (2023)					Not details
Mangin et al. (29)	Memory	Daily life activities, PEI, Mobility IPAQ (short)	Social life/support, Friendship Scale/Custom	Nutrition Screen II	Frailty, MTBQ, EQs 5D-5L, General health Edmonton Frail Scale, sleep 15-D, Recommended Oral Health Screening Questions, Community program and service use (adapted), Health Screening Questions, Smoking and alcohol Custom, TAP-Report (29)
Melis et al. (16)	MMSE	GARS-3	–	–	MOS
Metzelthin et al. (43)		Daily life performance, daily physical activity	Meaningful activities, adapting environment, social network and activities		Risk factors for developing disability
Monteserin et al. (33)	SPMSQ, 5-Yesavage Depression Scale	BI, Lawton IADL Falls	Gijon Social Scale	MNA-SF	Charlson Index, medication, perceived health status (one question), sensory evaluation (sight and hearing), Urinary incontinence
Montgomery and Fallis (17)	MMSE	ADL, IADL Home Care Rating System, TUG, Functional Reach	Social support Caregiver burden—Zarit Burden Scale, Caregiver Satisfaction Scale	–	Environmental assessment
Mueller et al. (34)	Mini-Cog PHQ-2	History of falls during past year; gait observation	–	Weight loss	Vision/hearing screening, osteoporosis
Orcel et al. (30)	DHP	ADL, DHP	DHP		DHP
Reuben et al. (35)					Upon request
Rockwood et al. (41)	MMSE Emotional health	BI, PSMS, IADL, Balance, mobility	Social support	Nutrition	Medications, service use, SQLI modified, self-rated health question
Romskaug et al. (28)	IQCODE, CDR ICD-10 criteria	Gait, falls mobility: gait problems; dizziness; walking aids; history of falling	–	MNA-SF, weight loss, reduced appetite, nausea, dyspepsia, BMI	Clinical tests, ECG, labs, Pain, Breathing, Hydration, urinary incontinence, voiding problems, diarrhea/constipation, Sleep, medications, clinical examination, Supplementary tests: blood pressure including orthostatic pulse rate, respiratory rate; ECG, blood analyses, drugs serum concentration, pharmacogenetic tests
Safari et al. (42)		Falls, mobility and balance	Function, social, environment		Physical health, Medication, Bone health, Care and support plan
Silverman et al. (18)	MMSE, CDR3 DIS	ADL, BI	–	–	Urinary and bowel Incontinence, Self- perceived health status
Sommers et al. (19)	–	Completed but not detailed	Home safety check	–	Clinical visit, health concerns, vital signs, health histories
Spoorenberg et al. (25)					No details
Stensvik et al. (26)	CDR CSDD, NPI-Q	PSMS	–	–	QUALID, CMAI

ADL, Activities of Daily Living; BANSS, Bedford Alzheimer Nursing Severity Scale; BI, Barthel Index; BPRS, Brief Psychiatric Rating Scale; BSI, Brief Symptom Inventory; CAPEBRS, CAPE behaviour rating scale; CBI, Caregiver Burden Inventory; CHSA, Canadian Study of Health Survey; COC, Continuity of Care Index; CSI, Computerized Severity Index; DBMA, Disease Burden Morbidity Assessment; DHP, Duke Health Profile; FBQ, Financial Benefits Questionnaire; FIM, Functional Independence Measure; FRT, Functional Reach Test; GARS-3, Groningen Activity Restriction Scale; GDS, Geriatric Depression Scale; GP, General Practitioner; IADL, Instrumental Activities of Daily Living; IPAQ, International Physical Activity Questionnaire; LSNS, Lubben Social Network Scale; MNA, Mini Nutritional Assessment; MMSE, Standardized Mini-Mental State Examination; MMS, Mini-Mental State; MOLST, Medical Outcomes of Life Sustaining Treatments; MOS, Medical Outcomes Study; MTBQ, Multimorbidity Treatment Burden Questionnaire; PEI, Patient Enablement Instrument; PGCMSR, Philadelphia Geriatric Center Morale Scale-Revised; PPI, Pressing Problem Index, PSMS, Physical Self-Maintenance Scale, PSQ, Patient Satisfaction Questionnaire; QAR, Quality Assurance Review Instrument; RAPA, Rapid Assessment of Physical Activity, SQLI, Spitzer Quality of Life Index; SPMSQ, Short Portable Mental Status Questionnaire; SSQ, Support Services Questionnaire; SSS, Satisfaction with Support Scale; TUG, Timed Up and Go test; US/SUS, Usability System Scale.

Most of the studies provide a detailed assessment using well-documented instruments (see details in Supplementary Table 4). In contrast, others refer to previously published protocols or do not explicitly specify the instruments used (3–5, 21, 25, 35, 36). In some cases, data are derived from standardized multidimensional assessments, such as the GRACE protocol (22) or the interRAI Community Health Assessment method (24).

## 4 Discussion

This systematic literature review provides an updated, in-depth analysis of CGAM in the primary care as a multidisciplinary process designed to assess older adults’ medical, functional, and social needs and develop a comprehensive, integrated, and personalized care plan. As recommended (44), this review was conducted by an evidence review team with both technical and clinical expertise, ensuring a rigorous approach. Particular attention was given to identifying the core composition of CGAM teams, including the number and expertise of professionals involved, as well as the instruments used to assess older adults people’s needs. The synthesis of evidence was based on 31 studies published between 1996 and 2026.

Our findings reveal substantial heterogeneity in CGAM team composition, particularly regarding the number of professionals involved and their specific roles. Most teams consisted of three to five members, a range that appears to balance the diverse needs of older adults with the available healthcare resources. However, the observed variability may suggest that CGAM interventions are adaptable to different clinical settings and contexts. While smaller teams may suffice in less complex cases, situations involving multimorbidity or frailty often necessitate larger, more specialized teams. A recent review by Kshatri and colleagues (2025) (45) highlighted significant variation in CGAM delivery models outside hospital settings, particularly regarding team composition. Although most models featured CGAM delivered by a nursing professional (9/22 studies) or a multidisciplinary team (6/22 studies), no specific recommendations were provided regarding the ideal number of team members. Further, findings from this review suggest that CGAM team composition and the extent of primary care physician involvement may vary according to geographic distribution reflecting underlying healthcare-system organization and resource availability. However, because the included evidence is predominantly derived from high-income countries, these patterns should be interpreted cautiously, as the current literature does not allow robust conclusions about the relative contribution of cultural versus economic/structural determinants to CGAM team configuration.

The importance of an interprofessional team approach has been widely recognized (46) as essential for addressing patients’ medical, functional, and social needs while aligning care with their personal goals. In this perspective, the involvement of various professionals working, ideally, in a coordinated manner is essential: physicians oversee medical treatment, nurses address comprehensive care needs, occupational therapists focus on activities of daily living and assistive devices, physiotherapists assess mobility and transfers, and social workers evaluate support systems and necessary interventions. The presence of a designated team leader to coordinate interventions across disciplines is reported as an essential factor (47).

This review identified nurses, geriatricians, and social workers as the most frequently involved professionals in CGAM, with additional specialists contributing to either an assessment or consultative role. Social workers were frequently identified as core team members, primarily responsible for strengthening links with community support services, as also reported by Arendts et al. (48). Their involvement underscores the necessity of integrating social care into CGAM to ensure that care plans address not only medical and functional aspects but also broader social determinants of health. Notably, the collaboration between CGAM teams and GPs has been consistently documented in studies examining CGAM implementation in primary care (6). Similarly, our findings indicate that GPs were involved in more than half of the included studies. Although they did not play a direct role in the assessment process, they contributed significantly to the design and implementation of individualized care plans, recommend actions, provide feedback, and confirm the care plan, underscoring their potential importance in multidisciplinary geriatric care. Finally, to ensure a comprehensive and nuanced understanding of an individual’s needs and to achieving fully integrated and coordinated care, it is crucial that older adults and their families are actively engaged in the CGAM process. They should be treated as equal partners in the assessment team, and their involvement should be systematically promoted (49). Although this aspect was not explicitly addressed in our review, several studies highlight and emphasize the value of home visits as a means to effectively integrate the feedback provided by the beneficiary of the care plan and their family. Further studies are needed to explore the degree and methods of users and family and/or caregivers’ involvement in the CGAM.

Consistent with previous findings (7, 50), the present review identified substantial variability in the instruments used to implement CGAM and assess individuals’ health and social needs. While numerous geriatric assessment instruments exist, their application and clinical utility vary depending on factors such as healthcare provider training, resource availability, and user characteristics. The assessment instruments employed in the studies reviewed addressed multiple dimensions of older adults’ health, including physical, cognitive, emotional, and social functions to support clinical decision-making and improve patient outcomes. Previous research has highlighted the wide variability of assessment instruments in integrated care programs (4, 7, 50, 51), which may reflect variations in CGAM team composition and hinder the establishment of a common language across healthcare settings. This lack of harmonization may translate into inconsistencies in assessment outcomes. In addition, heterogeneity may be further amplified by variability in what constitutes “standard care” across trials, with several studies reporting insufficient socio-demographic and clinical characterization of control groups, making it difficult to disentangle the effects of CGAM from differences in baseline care pathways. Additionally, the absence of consensus on the definition of frailty and the limited evidence on effective screening, diagnosis, and interventions may contribute to the observed variability.

Health information technology is widely recognized as a critical instrument for improving coordination and overall healthcare performance. However, this review found limited evidence of evidence-based assessment instruments that are digitally implemented and standardized across different care settings. One notable exception is the interRAI system (24), which has been implemented in Belgium, Switzerland, France, Ireland, Iceland, Finland, and New Zealand (52). Despite its potential benefits, several technical barriers hinder its widespread adoption. These include insufficient IT infrastructure and low interoperability when interRAI software is incompatible with existing healthcare systems (52). Although the interRAI system represents one of the most comprehensive and standardized approaches to multidimensional assessment, its implementation in primary care is challenged by the length of administration, the training required, and limited interoperability with existing electronic health records. These barriers may reduce feasibility in routine practice, despite the system’s potential to enhance multidisciplinary communication and decision-making.

The final and most critical step of CGAM is the development of personalized care plans but data regarding the implementation, monitoring, and adherence to care plan recommendations should be further investigated since CGAM effectiveness may depend not only on assessment of quality but also on organizational capacity to translate recommendations into sustained actions within patients’ living environments. Consistent with previous reviews, substantial variability persists in how multidisciplinary teams formulate and monitor personalized care plans, highlighting the need for clearer operational standards within primary care settings (50, 53). Although structured algorithms and pragmatic care pathways in geriatric and primary care settings have been proposed (51, 54), the evidence synthesized in this review does not provide sufficient granularity to translate these findings into a unified, evidence-based CGAM implementation model. It should be noted that while the implementation detail available in published studies may be incomplete, the recency of the update search is unlikely to be a major source of bias, as the synthesis is not limited to recent publications and includes the earlier randomized controlled trials already captured in rigorous reviews such as Briggs et al. (4)

Finally, the pattern of methodological quality across studies suggests a systematic tension between the complexity of CGAM as a multidimensional, team-based intervention and the feasibility of applying traditional randomized and blinded study designs in primary care and community settings. This may reflect not only methodological limitations, but also structural constraints intrinsic to evaluating complex care models. As a result, future CGAM research review may consider adopting less restrictive methodological criteria when selecting study designs, in order to capture and synthesize detailed descriptions of CGAM models regardless of whether they were primarily evaluated for effectiveness, thereby providing a more comprehensive understanding of CGAM structure and implementation across diverse care contexts.

## 5 Conclusion

This literature review highlights that, despite strong evidence supporting the effectiveness of CGAM, its implementation in primary care remains highly heterogeneous and not standardized. Persistent variability in team composition, assessment instruments, and care coordination models may reflect both contextual adaptation and unresolved structural challenges. Progress in CGAM research and practice should focus on defining minimum standards for CGAM delivery, improve pathways reporting, strengthening interdisciplinary coordination and developing scalable, implementation-ready models supported by digital tools.

## Footnotes

1
www.who.int/ictrp/en/

2
www.mcmasteroptimalaging.org/
