# Supplementary material for: Comprehensive geriatric assessment and management in primary care: a systematic literature review with a descriptive mapping of team composition and assessment instruments
Source: Front Public Health. 2026 Mar 20;14:1739380. doi: 10.3389/fpubh.2026.1739380 (PMC13047180; doi:10.3389/fpubh.2026.1739380)
Supplement: Supplementary file 1 [file Supplementary_file_1.docx]

[Search Strategies 2](#_Toc220422503)

[Table 1. Participants details 11](#_Toc220422504)

[Table 2. Inclusion and exclusion criteria of the included studies 12](#_Toc220422505)

[Table 3. Risk of bias 15](#_Toc220422506)

[Table 4. Details on CGAM instruments for each study 16](#_Toc220422507)

## Search Strategies

MEDLINE Proquest

including Epub Ahead of Print, In-Process & Other Non-Indexed Citations 1946 to Present. Search strategy from ProQuest. Limit search starting from 01 January 2020 to 31 December 2024

| Set# | Searched for |  | Results |
| --- | --- | --- | --- |
| S1 | (MESH.EXACT("Geriatric Assessment") OR MESH.EXACT("health services for the aged") OR tiab((GERIATRIC? OR ELDER* OR "OLD AGE" OR "OLD* ADULT?" OR SENIOR? OR "OLD* PATIENT?") NEAR/5 (ASSESS* OR EVALUAT* OR CONSULT*)) OR (MESH.EXACT("Health Status") AND MESH.EXACT.EXPLODE("Aged"))) | MEDLINE®, MEDLINE® | 109,360 |
| S2 | ((MESH.EXACT("Primary Health Care") OR MESH.EXACT("physicians, family") OR MESH.EXACT("physicians, primary care") OR MESH.EXACT("general practice") OR MESH.EXACT("general practitioners") OR MESH.EXACT("family practice") OR MESH.EXACT("practice patterns, physicians") OR MESH.EXACT("ambulatory care") OR MESH.EXACT("outpatient clinics, hospital") OR MESH.EXACT("community health centers") OR MESH.EXACT.EXPLODE("community health services") OR MESH.EXACT("community health planning") OR MESH.EXACT("community-based participatory research") OR MESH.EXACT("independent living") OR MESH.EXACT("day care, medical") OR MESH.EXACT("residential facilities") OR MESH.EXACT("assisted living facilities") OR MESH.EXACT("group homes") OR MESH.EXACT("halfway houses") OR MESH.EXACT("homes for the aged") OR MESH.EXACT.EXPLODE("nursing homes") OR tiab((communit* NEAR/3 (care OR healthcare OR service? OR network? OR based OR initiative* OR intervention* OR schem* OR participat* OR project* OR program* OR activit* OR partnership* OR action OR strategy*))) OR TIAB(primary NEAR/2 (care OR healthcare)) OR TIAB(("family practice" OR "family practiced" OR "family practices" OR "family practitioner" OR "family practitioners") OR ("family doctor" OR "family doctors") OR ("family physician" OR "family physicians") OR gp* OR ("general practice" OR "general practices" OR "general practitioner" OR "general practitioners")) OR TIAB(group? PRE/0 (home? OR living)) OR TIAB((home OR domicil*) NEAR/3 (care OR healthcare OR nurs* OR rehabilit* OR service OR services OR treatment? OR therapy OR therapies OR therapist? OR visiting OR visit?)) OR TIAB(residential NEAR/3 (care OR healthcare OR facilit*)) OR TIAB("day hospital?" OR (adult? OR elder* OR geriatric?) NEAR/2 ("day care" OR daycare)) OR TIAB(("halfway house" OR "halfway houses")) OR TIAB("respite care") OR TIAB("at home?" OR "own home?") OR TIAB(liv* NEAR/1 independent*))) | MEDLINE®, MEDLINE® | 1,258,897 |
| S3 | [S1] AND [S2] | MEDLINE®,  MEDLINE®  These databases are searched for part of your query. | 28,314 |
| S4 | TIAB(randomi?ed) OR AB(PLACEBO) OR TIAB(RANDOMLY) OR TI(TRIAL) OR MESH.EXACT("Randomized Controlled Trial as Topic") OR MESH.EXACT("Clinical Trials as Topic") | MEDLINE®, MEDLINE® | 1,513,659 |
| S5 | (RTYPE("Randomized Controlled Trial" OR "Controlled Clinical Trial" OR "Equivalence Trial" OR "Pragmatic Clinical Trial" ) OR DTYPE("Randomized Controlled Trial" OR "Controlled Clinical Trial" OR "Equivalence Trial" OR "Pragmatic Clinical Trial" )) | MEDLINE®, MEDLINE® | 710,009 |
| S6 | [S4] OR [S5] | MEDLINE®,  MEDLINE®  These databases are searched for part of your query. | 1,683,105 |
| S7 | MESH.EXACT.EXPLODE(animals) NOT MESH.EXACT(humans) | MEDLINE®, MEDLINE® | 5,217,998 |
| S8 | [S6] NOT [S7] | MEDLINE®,  MEDLINE®  These databases are searched for part of your query. | 1,553,258 |
| S9 | [S3] AND [S8] | MEDLINE®,  MEDLINE®  These databases are searched for part of your query. | 3,107 |
| S10 | ([S3] AND [S8]) AND pd(20200101-20241231) | MEDLINE®,  MEDLINE®  These databases are searched for part of your query | 372 |

Embase (Ovid)

1974 to Present. Limit search starting from 2020 to 2024

| No. | Query Results | Results |
| --- | --- | --- |
| #100 | #98 AND #99 | 519 |
| #99 | [2020-2024]/py | 8,089,241 |
| #98 | #85 AND #97 | 2,192 |
| #97 | #95 NOT #96 | 2,733,471 |
| #96 | 'animal'/exp NOT 'human'/de | 6,283,731 |
| #95 | #86 OR #87 OR #88 OR #89 OR #90 OR #91 OR #92 OR #93 OR #94 | 3,041,093 |
| #94 | 'randomised controlled trial'/de | 823,796 |
| #93 | 'randomised controlled trial'/de | 823,796 |
| #92 | 'single blind procedure'/de | 54,88 |
| #91 | 'crossover procedure'/de | 77,967 |
| #90 | assign*:ti,ab OR allocat*:ti,ab OR volunteer*:ti,ab OR placebo*:ti,ab | 1,315,820 |
| #89 | ((doubl* OR singl*) NEXT/1 blind*):ti,ab | 284,801 |
| #88 | crossover*:ti,ab OR 'cross over*':ti,ab | 130,425 |
| #87 | factorial*:ti,ab | 49,62 |
| #86 | random*:ti,ab | 2,066,550 |
| #85 | #56 AND #84 | 14,341 |
| #84 | #57 OR #58 OR #59 OR #60 OR #61 OR #62 OR #63 OR #64 OR #65 OR #66 OR #67 OR #68 OR #69 OR #70 OR #71 OR #72 OR #73 OR #74 OR #75 OR #76 OR #77 OR #78 OR #79 OR #80 OR #81 OR #82 OR #83 | 1,433,188 |
| #83 | (liv* NEAR/1 independent*):ti,ab | 8,821 |
| #82 | 'at home$':ti,ab OR 'own home$':ti,ab | 104,861 |
| #81 | 'respite care':ti,ab | 1,128 |
| #80 | 'halfway hous*':ti,ab | 318 |
| #79 | day hospital$':ti,ab OR (((adult$ OR elder* OR geriatric$) NEAR/2 ('day care' OR daycare)):ti,ab) | 9,634 |
| #78 | (residential NEAR/3 (care OR healthcare OR facilit*)):ti,ab | 10,391 |
| #77 | ((home OR domicil*) NEAR/3 (care OR healthcare OR nurs* OR rehabilit* OR service OR services OR treatment$ OR therapy OR therapies OR therapist$ OR visiting OR visit$)):ti,ab | 118,087 |
| #76 | (group$ NEXT/1 (home$ OR living)):ti,ab | 4,416 |
| #75 | 'family practi*':ti,ab OR 'family doctor*':ti,ab OR 'family physician*':ti,ab OR gp*:ti,ab OR 'general practi*':ti,ab | 468,937 |
| #74 | (primary NEAR/2 (care OR healthcare)):ti,ab | 243,985 |
| #73 | (communit* NEAR/3 (care OR healthcare OR service$ OR network$ OR based OR initiative* OR intervention* OR schem* OR participat* OR project* OR program* OR activit* OR partnership* OR action OR strategy*)):ti,ab | 218,719 |
| #72 | 'nursing home'/de | 66,405 |
| #71 | 'home for the aged'/de | 13,146 |
| #70 | 'halfway house'/de | 1,415 |
| #69 | 'assisted living facility'/de | 3,348 |
| #68 | 'residential home'/de | 8,441 |
| #67 | 'day care'/exp | 14,346 |
| #66 | 'independent living'/de | 8,077 |
| #65 | 'community care'/de | 67,151 |
| #64 | 'senior center'/de | 492 |
| #63 | 'community program'/de | 3,567 |
| #62 | 'health center'/de | 44,265 |
| #61 | 'outpatient department'/de | 98,166 |
| #60 | 'ambulatory care'/de | 45,471 |
| #59 | 'general practice'/de | 93,911 |
| #58 | 'general practitioner'/de | 124,433 |
| #57 | 'primary health care'/exp | 217,779 |
| #56 | #51 OR #52 OR #55 | 65,026 |
| #55 | #53 AND #54 | 5,572 |
| #54 | 'evaluation and follow up'/exp | 3,711,657 |
| #53 | 'geriatrics'/de | 51,016 |
| #52 | ((geriatric$ OR elder* OR 'old age' OR 'old* adult$' OR senior$ OR 'old* patient$') NEAR/5 (assess* OR evaluat* OR consult*)):ti,ab | 48,858 |
| #51 | 'geriatric assessment'/exp OR 'geriatric assessment' | 24,829 |
| #50 | #48 AND #49 | 519 |
| #49 | [2020-2024]/py | 8,089,241 |
| #48 | #35 AND #47 | 2,192 |
| #47 | #45 NOT #46 | 2,733,471 |
| #46 | 'animal'/exp NOT 'human'/de | 6,283,731 |
| #45 | #36 OR #37 OR #38 OR #39 OR #40 OR #41 OR #42 OR #43 OR #44 | 3,041,093 |
| #44 | 'randomised controlled trial'/de | 823,796 |
| #43 | 'randomised controlled trial'/de | 823,796 |
| #42 | 'single blind procedure'/de | 54,88 |
| #41 | 'crossover procedure'/de | 77,967 |
| #40 | assign*:ti,ab OR allocat*:ti,ab OR volunteer*:ti,ab OR placebo*:ti,ab | 1,315,820 |
| #39 | ((doubl* OR singl*) NEXT/1 blind*):ti,ab | 284,801 |
| #38 | crossover*:ti,ab OR 'cross over*':ti,ab | 130,425 |
| #37 | factorial*:ti,ab | 49,62 |
| #36 | random*:ti,ab | 2,066,550 |
| #35 | #6 AND #34 | 14,341 |
| #34 | #7 OR #8 OR #9 OR #10 OR #11 OR #12 OR #13 OR #14 OR #15 OR #16 OR #17 OR #18 OR #19 OR #20 OR #21 OR #22 OR #23 OR #24 OR #25 OR #26 OR #27 OR #28 OR #29 OR #30 OR #31 OR #32 OR #33 | 1,433,188 |
| #33 | (liv* NEAR/1 independent*):ti,ab | 8,821 |
| #32 | 'at home$':ti,ab OR 'own home$':ti,ab | 104,861 |
| #31 | 'respite care':ti,ab | 1,128 |
| #30 | 'halfway hous*':ti,ab | 318 |
| #29 | 'day hospital$':ti,ab OR (((adult$ OR elder* OR geriatric$) NEAR/2 ('day care' OR daycare)):ti,ab) | 9,634 |
| #28 | (residential NEAR/3 (care OR healthcare OR facilit*)):ti,ab | 10,391 |
| #27 | ((home OR domicil*) NEAR/3 (care OR healthcare OR nurs* OR rehabilit* OR service OR services OR treatment$ OR therapy OR therapies OR therapist$ OR visiting OR visit$)):ti,ab | 118,087 |
| #26 | (group$ NEXT/1 (home$ OR living)):ti,ab | 4,416 |
| #25 | 'family practi*':ti,ab OR 'family doctor*':ti,ab OR 'family physician*':ti,ab OR gp*:ti,ab OR 'general practi*':ti,ab | 468,937 |
| #24 | (primary NEAR/2 (care OR healthcare)):ti,ab | 243,985 |
| #23 | (communit* NEAR/3 (care OR healthcare OR service$ OR network$ OR based OR initiative* OR intervention* OR schem* OR participat* OR project* OR program* OR activit* OR partnership* OR action OR strategy*)):ti,ab | 218,719 |
| #22 | 'nursing home'/de | 66,405 |
| #21 | 'home for the aged'/de | 13,146 |
| #20 | 'halfway house'/de | 1,415 |
| #19 | 'assisted living facility'/de | 3,348 |
| #18 | 'residential home'/de | 8,441 |
| #17 | 'day care'/exp | 14,346 |
| #16 | 'independent living'/de | 8,077 |
| #15 | 'community care'/de | 67,151 |
| #14 | 'senior center'/de | 492 |
| #13 | 'community program'/de | 3,567 |
| #12 | 'health center'/de | 44,265 |
| #11 | 'outpatient department'/de | 98,166 |
| #10 | 'ambulatory care'/de | 45,471 |
| #9 | 'general practice'/de | 93,911 |
| #8 | 'general practitioner'/de | 124,433 |
| #7 | 'primary health care'/exp | 217,779 |
| #6 | #1 OR #2 OR #5 | 65,026 |
| #5 | #3 AND #4 | 5,572 |
| #4 | 'evaluation and follow up'/exp | 3,711,657 |
| #3 | 'geriatrics'/de | 51,016 |
| #2 | ((geriatric$ OR elder* OR 'old age' OR 'old* adult$' OR senior$ OR 'old* patient$') NEAR/5 (assess* OR evaluat* OR consult*)):ti,ab | 48,858 |
| #1 | 'geriatric assessment'/exp OR 'geriatric assessment' | 24,829 |

The Cochrane Library (Wiley)

Limit search starting from April 2020 to May 2024

| Query | Search String | Results |
| --- | --- | --- |
| #1 | [mh "geriatric assessment"] | 263 |
| #2 | [mh "health services for the aged"] | 26 |
| #3 | ((geriatric? or elder* or (old next age) or (old* next adult?) or senior? or (old* next patient?)) near/5 (assess* or evaluat* or consult*)):ti,ab | 1544 |
| #4 | [mh "health status"] | 15962 |
| #5 | [mh aged] | 28260 |
| #6 | #4 and #5 | 3671 |
| #7 | (#1 or #2 or #3 or #6) | 5030 |
| #8 | [mh "primary health care"] or [mh "physicians, family"] or [mh "physicians, primary care"] or [mh "general practice"] or [mh "general practitioners"] or [mh "family practice"] or [mh "practice patterns, physicians'"] or [mh "ambulatory care"] or [mh "outpatient clinics, hospital"] or [mh "community health centers"] or [mh "community health services"] or [mh "community health planning"] or [mh "community-based participatory research"] or [mh "independent living"] or [mh "day care, medical"] or [mh "residential facilities"] or [mh "assisted living facilities"] or [mh "group homes"] or [mh "halfway houses"] or [mh "homes for the aged"] or [mh "nursing homes"] | 7250 |
| #9 | (communit* near/3 (care or healthcare or service? or network? or based or initiative* or intervention* or schem* or participat* or project* or program* or activit* or partnership* or action or strategy*)):ti,ab | 7210 |
| #10 | (primary near/2 (care or healthcare)):ti,ab | 8578 |
| #11 | (family next practi* or family next doctor* or family next physician* or gp* or general next practi*):ti,ab | 6923 |
| #12 | (group? next (home? or living)):ti,ab | 19456 |
| #13 | ((home or domicil*) near/3 (care or healthcare or nurs* or rehabilit* or service or services or treatment? or therapy or therapies or therapist? or visiting or visit?)):ti,ab | 5452 |
| #14 | (residential near/3 (care or healthcare or facilit*)):ti,ab | 416 |
| #15 | ((adult? or elder* or geriatric?) near/2 ((day next care) or daycare)):ti,ab | 24 |
| #16 | ((halfway next hous*) or (respite next care) or (day next hospital?)):ti,ab | 229 |
| #17 | (at next home? or own next home?):ti,ab | 20954 |
| #18 | (liv* near/1 independent*):ti,ab | 353 |
| #19 | {or #8-#18} | 49071 |
| #20 | #7 and #19 | 1677 |

CINAHL (EBSCO)

Limit search starting from 01 April 2020 to 31 May 2024

| No. | Query | Limiter/Expanders | Results |
| --- | --- | --- | --- |
| S1 | (MH 'Geriatric Assessment+') | Apply equivalent subjects | 18920 |
| S2 | 'Health Services for the Aged' | Apply equivalent subjects | 5246 |
| S3 | TI ((geriatric? or elder* or old age or old* adult? or senior? or old* patient?) N5 (assess* or evaluat* or consult*)) OR AB ((geriatric? or elder* or old age or old* adult? or senior? or old* patient?) N5 (assess* or evaluat* or consult*)) | Apply equivalent subjects | 18392 |
| S4 | (MH 'Health Status+') | Apply equivalent subjects | 143078 |
| S5 | (MH 'Aged+') | Apply equivalent subjects | 963981 |
| S6 | S4 AND S5 | Apply equivalent subjects | 46514 |
| S7 | S1 OR S2 OR S3 OR S6 | Apply equivalent subjects | 78750 |
| S8 | (MH 'Primary Health Care') | Apply equivalent subjects | 74831 |
| S9 | (MH 'Physicians, Family') | Apply equivalent subjects | 23740 |
| S10 | (MH 'Family Practice') | Apply equivalent subjects | 26557 |
| S11 | (MH 'Practice Patterns') | Apply equivalent subjects | 13633 |
| S12 | (MH 'Ambulatory Care') | Apply equivalent subjects | 14037 |
| S13 | (MH 'Outpatient Service') | Apply equivalent subjects | 12246 |
| S14 | (MH 'Ambulatory Care Facilities+') | Apply equivalent subjects | 18018 |
| S15 | (MH 'Community Health Centers') | Apply equivalent subjects | 7357 |
| S16 | (MH 'Community Health Services') | Apply equivalent subjects | 26861 |
| S17 | (MH 'Senior Centers') | Apply equivalent subjects | 158 |
| S18 | (MH 'Home Health Care') | Apply equivalent subjects | 26923 |
| S19 | (MH 'Home Visits') | Apply equivalent subjects | 6794 |
| S20 | (MH 'Respite Care') | Apply equivalent subjects | 1491 |
| S21 | 'Housing for the Elderly' | Apply equivalent subjects | 2533 |
| S22 | (MH 'Skilled Nursing Facilities') | Apply equivalent subjects | 4866 |
| S23 | (MH 'Nursing Homes') | Apply equivalent subjects | 26952 |
| S24 | (MH 'Halfway Houses') | Apply equivalent subjects | 220 |
| S25 | (MH 'Residential Facilities') | Apply equivalent subjects | 5503 |
| S26 | (MH 'Community Living+') | Apply equivalent subjects | 26543 |
| S27 | (MH 'Day Care') | Apply equivalent subjects | 2953 |
| S28 | TI (communit* N3 (care or healthcare or service? or network? or based or initiative* or intervention* or schem* or participat* or project* or program* or activit* or partnership* or action or strategy*)) OR AB (communit* N3 (care or healthcare or service? or network? or based or initiative* or intervention* or schem* or participat* or project* or program* or activit* or partnership* or action or strategy*)) | Apply equivalent subjects | 105528 |
| S29 | TI (primary N2 (care or healthcare)) OR AB (primary N2 (care or healthcare)) | Apply equivalent subjects | 105078 |
| S30 | TI (family practi* or family doctor* or family physician* or gp* or general practi*) OR AB (family practi* or family doctor* or family physician* or gp* or general practi*) | Apply equivalent subjects | 67029 |
| S31 | TI (group? N0 (home? or living)) OR AB (group? N0 (home? or living)) | Apply equivalent subjects | 1430 |
| S32 | TI ((home or domicil*) N3 (care or healthcare or nurs* or rehabilit* or service or services or treatment? or therapy or therapies or therapist? or visiting or visit?)) OR AB ((home or domicil*) N3 (care or healthcare or nurs* or rehabilit* or service or services or treatment? or therapy or therapies or therapist? or visiting or visit?)) | Apply equivalent subjects | 75813 |
| S33 | TI (residential N3 (care or healthcare or facilit*)) OR AB (residential N3 (care or healthcare or facilit*)) | Apply equivalent subjects | 7332 |
| S34 | TI (day hospital? or ((adult? or elder* or geriatric?) N2 (day care or daycare))) OR AB (day hospital? or ((adult? or elder* or geriatric?) N2 (day care or daycare))) | Apply equivalent subjects | 1867 |
| S35 | TI (halfway hous* or respite care or at home? or own N0 home?) OR AB (halfway hous* or respite care or at home? or own N0 home?) | Apply equivalent subjects | 174340 |
| S36 | TI (liv* N1 independent*) OR AB (liv* N1 independent*) | Apply equivalent subjects | 5035 |
| S37 | S8 OR S9 OR S10 OR S11 OR S12 OR S13 OR S14 OR S15 OR S16 OR S17 OR S18 OR S19 OR S20 OR S21 OR S22 OR S23 OR S24 OR S25 OR S26 OR S27 OR S28 OR S29 OR S30 OR S31 OR S32 OR S33 OR S34 OR S35 OR S36 | Apply equivalent subjects | 559858 |
| S38 | S7 AND S37 | Apply equivalent subjects | 22454 |
| S39 | PT randomized controlled trial | Apply equivalent subjects | 156834 |
| S40 | PT clinical trial | Apply equivalent subjects | 114227 |
| S41 | TI (randomis* or randomiz* or randomly) OR AB (randomis* or randomiz* or randomly) | Apply equivalent subjects | 396633 |
| S42 | (MH 'Clinical Trials+') | Apply equivalent subjects | 356346 |
| S43 | (MH 'Random Assignment') | Apply equivalent subjects | 85178 |
| S44 | S39 OR S40 OR S41 OR S42 OR S43 | Apply equivalent subjects | 579465 |
| S45 | S38 AND S44 | Limiters  - PublicationDate: 20200401-20240531  Expanders  - Applyequivalent subjects  Search modes  -Boolean/Phrase | 556 |

ClinicalTrials.gov

Limit search starting from 01 January 2020 to 23 May 2024

| Other terms | primary care OR general practice OR family practice |
| --- | --- |
| Intervention | geriatric assessment |
| Study type | interventional studies |

WHO International Clinical Trials Registry Platform (ICTRP)

Search starting from the inception to 23 May 2024

Search terms

geriatric assessment AND primary care

geriatric assessment AND general practice

geriatric assessment AND family practice

Mc Master aging portal

Search starting from the inception to 23 May 2024

| Search terms | Results |
| --- | --- |
| geriatric assessment AND primary care | 12 |
| geriatric assessment AND general practice | 2 |
| geriatric assessment AND family practice | 25 |

Scopus citing and cited references

Cited reference search on 30 included studies, 26 July 2024

| Included studies | Citing | Cited by |
| --- | --- | --- |
| Bernabei R, Landi F, Gambassi G, Sgadari A, Zuccala G, Mor V, et al. Randomised trial of impact of model of integrated care and case management for older people living in the community. BMJ 1998;316(7141):1348-51. | 18 | 368 |
| Boult C, Boult LB, Morishita L, Dowd B, Kane RL, Urdangarin CF. A randomized clinical trial of outpatient geriatric evaluation and management. Journal of the American Geriatrics Society 2001;49:351-9. | 50 | 217 |
| Brazil K, Cardwell C, Carter G, Clarke M, Corry DAS, Fahey T, Gillespie P, Hobbins A, McGlade K, O'Halloran P, O'Neill N, Wallace E, Doyle F. Anticipatory care planning for community-dwelling older adults at risk of functional decline: a feasibility cluster randomized controlled trial. BMC Geriatr. 2022 May 25;22(1):452 | 38 | 1 |
| Clarkson P, Venables D, Hughes J, Burns A, Challis D. Integrated specialist assessment of older people and predictors of care-home admission. Psychological Medicine 2006;36:1011-21. | 64 | 12 |
| Counsell SR, Callahan CM, Clark DO, Tu W, Buttar AB, Stump TE, et al. Geriatric care management for low income seniors: a randomized controlled trial. JAMA 2007;298(22):2623-33. | 56 | 414 |
| De Luca, R., Torrisi, M., Bramanti, A., Maggio, M. G., Anchesi, S., Andaloro, A., Caliri, S., De Cola, M. C., & Calabrò, R. S. (2021). A multidisciplinary Telehealth approach for community dwelling older adults. Geriatric Nursing (New York, N.Y.), 42(3), 635–642 | 75 | 6 |
| Di Pollina L, Guessous I, Petoud V, Combescure C, Buchs B, Schaller P, et al. Integrated care at home reduces unnecessary hospitalizations of community dwelling frail older adults: a prospective controlled trial. BMC Geriatrics 2017;17:53. | 51 | 83 |
| Ekdahl AW, Alwin J, Eckerblad J, Husberg M, Jaarsma T, Lindh A, et al. Long-term evaluation of the Ambulatory Geriatric Assessment: a Frailty Intervention Trial (AGe-FIT): clinical outcomes and total costs after 36 months. Journal of the American Medical Director's Association 2016;17(3):263-8. | 30 | 52 |
| Engelhardt JB, Toseland RW, O'Donnell JC, Richie JT, Jue D, Banks S. The effectiveness and efficiency of outpatient geriatric evaluation and management. Journal of the American Geriatrics Society 1996;44:847-56. | 56 | 74 |
| Fairhall N, Sherrington C, Kurrle SE, Lord SR, Lockwood K, Howard K, et al. Economic evaluation of a multifactorial, interdisciplinary intervention versus usual care to reduce frailty in frail older people. Journal of the American Medical Director's Association 2015;16(1):41-8. | 22 | 99 |
| Federman, A. D., Brody, A., Ritchie, C. S., Egorova, N., Arora, A., Lubetsky, S., Goswami, R., Peralta, M., Reckrey, J. M., Boockvar, K., Shah, S., Ornstein, K. A., Leff, B., DeCherrie, L., & Siu, A. L. (2023). Outcomes of home-based primary care for homebound older adults: A randomized clinical trial. Journal of the American Geriatrics Society, 71(2), 443–454 | 39 | 11 |
| Fristedt S, Nystedt P, Skogar O. Mobile geriatric teams – a cost-effective way of improving patient safety and reducing traditional healthcare utilization among the frail elderly? A randomized controlled trial. Clinical Interventions in Aging 2019;14:1911-24. | 27 | 11 |
| Hoogendijk EO, van der Horst HE, van de Ven PM, Twisk JW, Deeg DJ, Frijters DH, et al. Effectiveness of a Geriatric Care Model for frail older adults in primary care: results from a stepped wedge cluster randomised trial. European Journal of Internal Medicine 2016;28:43-51. | 46 | 57 |
| Imhof L, Naef R, Wallhagen M, Schwarz J, Mahrer-Imhof R. Effects of an advanced practice nurse in home health consultation program for community-dwelling persons aged 80 and older. Journal of the American Geriatrics Society 2012;60(12):2223-31. | 57 | 60 |
| Li C-M, Chen C-Y, Li C-Y, Wang W-D, Wu S-C. The effectiveness of a comprehensive geriatric assessment intervention program for frailty in community-dwelling older people: a randomised, controlled trial. Archives of Gerontology and Geriatrics 2010;50 Suppl 1:S39-S42. | 33 | 99 |
| Lyndon, H., Latour, J. M., Marsden, J., & Kent, B. (2023). A nurse-led comprehensive geriatric assessment intervention in primary care: A feasibility cluster randomized controlled trial. Journal of Advanced Nursing, 79(9), 3473–3486 | 59 | 0 |
| Mangin, D., Lamarche, L., Oliver, D., Blackhouse, G., Bomze, S., Borhan, S., Carr, T., Clark, R., Datta, J., Dolovich, L., Gaber, J., Forsyth, P., Howard, M., Marentette-Brown, S., Risdon, C., Talat, S., Tarride, J.-É., Thabane, L., Valaitis, R., & Price, D. (2023). Health TAPESTRY Ontario: A Multi-Site Randomized Controlled Trial Testing Implementation and Reproducibility. Annals of Family Medicine, 21(2), 132–142 | 33 | 2 |
| Melis RJ, van Eijken MI, Teerenstra S, van Achterberg T, Parker SG, Borm GF, et al. A randomized study of a multidisciplinary program to intervene on geriatric syndromes in vulnerable older people who live at home (Dutch EASYcare Study). Journals of Gerontology. Series A, Biological Sciences and Medical Sciences 2008;63(3):283-90. | 35 | 120 |
| Metzelthin SF, van Rossum E, de Witte LP, Ambergen AW, Hobma SO, Sipers W, et al. Effectiveness of interdisciplinary primary care approach to reduce disability in community dwelling frail older people: cluster randomised controlled trial. BMJ 2013;347:f5264. | 48 | 132 |
| Monteserin R, Brotons C, Moral I, Altimir S, San Jose A, Santaeugenia S, et al. Effectiveness of a geriatric intervention in primary care: a randomised clinical trial. Family Practice 2010;27:239-45. | 25 | 70 |
| Montgomery PR, Fallis WM. South Winnipeg Integrated Geriatric Program (SWING): a rapid community-response program for the frail elderly. Canadian Journal on Aging 2003;22(3):275-81. | 21 | 14 |
| Mueller, Y., Schwarz, J., Monod, S., Locatelli, I., & Senn, N. (2021). Use of standardized brief geriatric evaluation compared with routine care in general practice for preventing functional decline: A pragmatic cluster-randomized trial. CMAJ: Canadian Medical Association Journal = Journal de l’Association Medicale Canadienne, 193(33), E1289–E1299 | 43 | 1 |
| Reuben DB, Frank JC, Hirsch SH, McGuigan KA, Maly RC. A randomized clinical trial of outpatient comprehensive geriatric assessment coupled with in intervention to increase adherence to recommendations. Journal of the American Geriatrics Society 1999;47:269-76. | 51 | 216 |
| Rockwood K, Stadnyk K, Carver D, MacPherson KM, Beanlands HE, Powell C, et al. A clinimetric evaluation of specialized geriatric care for rural dwelling, frail older people. Journal of the American Geriatrics Society 2000;48(9):1080-5. | 40 | 73 |
| Romskaug, R., Skovlund, E., Straand, J., Molden, E., Kersten, H., Pitkala, K. H., Lundqvist, C., & Wyller, T. B. (2020). Effect of Clinical Geriatric Assessments and Collaborative Medication Reviews by Geriatrician and Family Physician for Improving Health-Related Quality of Life in Home-Dwelling Older Patients Receiving Polypharmacy: A Cluster Randomized Clinical Trial. JAMA Internal Medicine, 180(2), 181–189 | 34 | 59 |
| Safari, R., Jackson, J., & Boole, L. (2023). Comprehensive geriatric assessment delivered by advanced nursing practitioners within primary care setting: A mixed-methods pilot feasibility randomised controlled trial. BMC Geriatrics, 23(1), 513 | 32 | 0 |
| Silverman M, Musa D, Martin DC, Lave JR, Adams J, Ricci EM. Evaluation of outpatient geriatric assessment: a randomized multi-site trial. Journal of the American Geriatrics Society 1995;43(7):733-40. | 38 | 100 |
| Sommers LS, Marton KI, Barbaccia JC, Randolph J. Physician, nurse, and social worker collaboration in primary care for chronically ill seniors. Archives of Internal Medicine 2000;160(12):1825-33. | 51 | 222 |
| Spoorenberg SL, Wynia K, Uittenbroek RJ, Kremer HP, Reijneveld SA. Effects of a population-based, person-centred and integrated care service on health, wellbeing and self management of community-living older adults: a randomized controlled trial on Embrace. PLoS One 2018;13(1):e0190751. | 65 | 44 |
| Stensvik, G.-T., Helvik, A.-S., Haugan, G., Steinsbekk, A., Salvesen, Ø., & Nakrem, S. (2022). The short-term effect of a modified comprehensive geriatric assessment and regularly case conferencing on neuropsychiatric symptoms in nursing homes: A cluster randomized trial. BMC Geriatrics, 22(1), 316 | 53 | 2 |

## Table 1. Participants details

|  | | | | |
| --- | --- | --- | --- | --- |
|  |  | Chronological age | | |
|  |  | Inclusion criteria |  | Participant’s age |
|  |  |  |  | m(sd) |
| Bernabei, 1998 |  | ≥ 65 |  | 80.7 (7.1) |
| Boult, 2001 |  | ≥ 70 |  | 78.8 (5.3) |
| Brazil, 2022 |  | ≥ 70 yr |  | 79.2 (5.4) |
| Clarkson, 2006 |  | Not specified |  | 82.0 (7) |
| Counsell, 2007 |  | ≥ 65 |  | 71.8 (5.6) |
| De Luca, 2021 |  | >65 |  | 77.4 (9.1) |
| Di Pollina, 2017 |  | ≥ 60 |  | 81.8 (8.2) |
| Ekdahl, 2016 |  | ≥ 65 |  | 82.3 (4.6) |
| Engelhardt, 1996 |  | ≥ 55 |  | 71.70 (6.8) |
| Fairhall, 2015 |  | ≥ 70 |  | 83.4 (5.81), |
| Federman, 2023 |  | ≥ 65 |  | 82.0 (8.8) |
| Fristedt, 2019 |  | ≥ 75 |  | 84.0 (5.1) |
| Hoogendijk, 2016 |  | ≥ 65 |  | 80.5 (7.5) |
| Imhof, 2012 |  | ≥ 80 |  | 85.0 (4) |
| Li, 2010 |  | ≥ 65 |  | 78.4 (8.2) |
| Lyndon, 2023 |  | ≥ 65 |  | 80.0 (7.3) |
| Mangin, 2023 |  | ≥ 70 |  | 77.5 (5.8) |
| Melis, 2008 |  | ≥ 70 |  | 81.7 (5.9) |
| Metzelthin, 2013 |  | ≥ 70 |  | 77.49 (5.28) |
| Monteserin, 2010 |  | ≥ 75 |  | 80.3 (-.--) |
| Montgomery, 2003 |  | ≥ 65 |  | 81.4 (7.2) |
| Mueller, 2021 |  | ≥ 75 |  | 82.7 (4.7) |
| Orcel, 2024 |  | ≥ 70 |  | 82 (77.1–86.5 age range) |
| Reuben, 1999 |  | ≥ 65 |  | 75.8 (6.1) |
| Rockwood, 2000 |  | Not specified |  | 81.4 (7.2) |
| Romskaug, 2019 |  | ≥ 70 |  | 82.2 (7.6) |
| Safari, 2023 |  | ≥ 65 |  | 82.0 (7.90) |
| Silverman, 1995 |  | ≥ 65 |  | 74.6 (7.46) |
| Sommers, 2000 |  | ≥ 65 |  | 78 (6.8) |
| Spoorenberg, 2018 |  | ≥ 75 |  | 80.6 (4.5) |
| Stensvik, 2022 |  | Not specified |  | 85.5 (8.3) |

## Table 2. Inclusion and exclusion criteria of the included studies

| Studies | Inclusion criteria | Exclusion criteria |
| --- | --- | --- |
| Bernabei, 1998 | - ≥ 65 yr - receiving home care because of multiple geriatric conditions | NA |
| Boult, 2001 | - ≥ 70 yr - medicare beneficiaries - high-risk of hospital admission defined by PRQ score | - living in nursing home - illness requiring frequent physician visits - communication barriers (cognitive impairment, deafness, inability to speak English) - restrictive insurance |
| Brazil, 2022 | - ≥ 70 yr enrolled in GMS/NHS primary care - multimorbidity (defined as ≥ 2 chronic medical conditions) - taking ≥ 4 regular medications - able to complete questionnaires in English | - received palliative care - cognitive impairment (Mini-Mental State Examination score of 20 or less) - psychosis - homelessness or long-term inpatient or nursing home care |
| Clarkson, 2006 | - older - community - dwelling - high levels of home care - at risk of requiring nursing home care | NA |
| Counsell, 2007 | - ≥ 65 yr - ≥ 1 visit to a primary care clinician within the past 12 mo - an income less than 200% of the federal poverty level | - nursing home resident - receiving dialysis - severe hearing loss - no access to phone - severe cognitive impairment at SPMSQ |
| De Luca, 2021 | - 65 > yr - affected by ≥ chronic medical or neuropsychiatric condition | - severe medical or psychiatric illness - severe cognitive deficits (MMSE < 15) |
| Di Pollina, 2017 | - ≥ 60 yr - frail - followed by a primary care physician, who had prescribed Home Visiting Nursing services center | - no frailty criteria - unable to give consent |
| Ekdahl, 2016 | - ≥ 65 yr - community dwelling - inpatient hospital care ≥ 3 in the previous 12 mo - ≥ 3 concomitant medical diagnoses | NA |
| Engelhardt, 1996 | - ≥ 55 yr - veterans - ≥ 10 clinic visits in the 12 mo - 2 ≥ ADL or IADL impairments | - hospitalised for psychiatric diagnoses in the 12 mo - severe cognitive impairment (SPMSQ) - received care within a year prior in outpatient clinics-interdisciplinary care; home-based primary care, adult day health care, inpatient geriatric evaluation and management unit; residing in nursing home; receiving the majority of their care in the previous year from non-VA providers |
| Fairhall, 2015 | - ≥ 70 yr - CHS criteria for frailty | - living in nursing home - severe cognitive impairment (MMSE < 19) - life expectancy < 1 year |
| Federman, 2023 | - ≥ 65 yr - homebound - ≥1 hospitalization in the prior 12 mo - need for assistance with ≥2 activities of daily living, and leaving the home ≤2 times each week* | - NA |
| Fristedt, 2019 | - ≥ 75 yr - frail - community-dwelling - 3 ≥ chronic dg - 6 ≥ pharmaceutical drugs - ≥ 3 hospital stays (> 24 h) over the last 6 mo | - living in nursing home - admissions for surgery rather than medical issue |
| Hoogendijk, 2016 | - ≥ 65 yr - frail (PRISMA-7 Score ≥3) | - death - dementia symptoms - moved to nursing home |
| Imhof, 2012 | - ≥ 80 years - community-dwelling | - end of life - major psychiatric diagnosis - severe cognitive impairment (Clinical Dementia Rating Scale) |
| Li, 2010 | - ≥ 65 yr - prefrail or frail (defined by Fried Criteria) | - bedridden - receiving home care - life expectancy < 6 months - difficulty in verbal communication |
| Lyndon, 2023 | - ≥ 65 yr - moderately and severely frail patients (frailty confirmed by PRISMA7 instrument, Raiche et al., 2008) - living in own home/supported living accommodation | - received palliative care with limited life expectancy - lack of mental capacity to give informed consent; |
| Mangin, 2023 | - ≥ 70 yr - rostered to participating family physicians permanently | - resided in a long-term care facility - factors that prevented participants from completing surveys (eg, severe dementia) |
| Melis, 2008 | - ≥ 70 yr - community - dwelling - current health problem related to cognition, mood, mobility/falls or malnutrition - 1 of the following: MMSE < 27, GARS-3 Score ≥ 25, MOS-20 Mental Health Subscale Score < 76 | - acutely unwell, requiring urgent medical attention - moderate-to-severe dementia or MMSE < 20 - receiving forms of intermediate care/health care from social worker/geriatrician - on waiting list for nursing home - life expectancy < 6 moo |
| Metzelthin, 2013 | - ≥ 70 yr - frail (GFI >= 5) | - terminally ill - bedridden - severe cognitive or psychological impairment - unable to communicate |
| Monteserin, 2010 | - ≥ 75 yr - ≥ 2 of the following conditions: - ≥ 85 years - Gijon Social Scale > 9 - Pfeiffer Test score ≥ 2 - Charlson Comorbidity Index score ≥ 2 - Yesavage Depression Scale score ≥ 1 - Barthel Index score ≥ 91 - Mini Nutritional Assessment score ≥ 12 - polymedication - > 1 fall in last 6 mo - urinary incontinence | - terminal diagnosis - living in nursing home - severe cognitive impairment - difficulty accessing primary care center |
| Montgomery, 2003 | - ≥ 65 yr - multiple problems, requiring coordinated follow-up home care | - well elderly and clients with single-issue problems readily dealt with by primary care providers |
| Mueller, 2021 | - ≥ 75 yr - home-living - ≥ 2 visits at GP in the previous year - able to understand French | - geriatric or specialized memory consultation in the 3 mo before recruitment |
| Orcel, 2024 | - ≥ 70 yr - long-term health condition or an unplanned   hospital admission in the previous 3 months  consultation with their usual GP or another GP in the same  investigating centre | - estimated life expectancy < 12 months - inability to speak or understand French - residence in an institutional - absence of health insurance coverage |
| Reuben, 1999 | - ≥ 65 yr - failed a screening assessment for at least 1 of 4 conditions (falls, urinary incontinence, depressive symptoms or functional impairment) | - not speaking English - no phone - no primary care physician - dementia or MMSE < 24 - mental, emotional, physical disorders limiting to complete the questionnaires |
| Rockwood, 2000 | - older - community - dwelling - frail (Rockwood et al., 1994) - rural practitioners | - NA |
| Romskaug, 2019 | - ≥ 70 yr - home - dwelling - ≥7 systemic medications regularly, administered by the home nursing service | - expected to die/become permanently institutionalized within 6 mo - Moderate/severe dementia (CDRS > 1) - Life expectancy ≤ 6 mo |
| Safari, 2023 | - ≥ 65 yr - eFI score of ≥ 0.12 showing mild, moderate, or severe levels of frailty (TUG test, Rockwood and PRISMA-7) | - not clinically judged as frail |
| Silverman, 1995 | - ≥ 65 yr - noninstitutionalized experiencing instability or change in their health status in previous 6 mo - subjects 60<aged >65 yr if met other criteria and need for care - change in health status defined as: ≥ 1 specific indicators (risk factors for institutionalization) or need for intervention to deal with serious deterioration | - too healthy - terminal illness - schizophrenia - resided in a nursing home |
| Sommers, 2000 | - ≥ 65 yr - ≥ 1 visit(s) with primary care physician in prior 4 mo - spoke English - independent in daily living: walking, transferring, toileting, and feeding - unable to carry out ≥ 1 instrumental activity of daily living: getting around outside the home, meal preparation and household chores, taking medications, use of the telephone, money management - under treatment for ≥ 2 chronic conditions   if both chronic conditions were stable, having ≥1 health risk (sedentary lifestyle, hyperlipidemia, obesity, smoking, alcoholism, social isolation, depression, anxiety)   - not under therapy for metastatic disease, Alzheimer, or dementias - not terminally ill - not residing in nursing home |  |
| Spoorenberg, 2018 | - ≥ 75 yr - living at home or in a home for the elderly | - long-term admission to a nursing home (not just for rehabilitation) |
| Stensvik, 2022 | - residents in regular care unit with long-term stay - residential time ≥ 60 dys - life expectancy ≥ 6 mo | - special dementia care units with enhanced staffing |

## Table 3. Risk of bias

|  | Jajad Score | Methodological quality category |
| --- | --- | --- |
| Bernabei, 1998 | 4 | Adequate -Higher quality (≥3) |
| Boult, 2001 | 2 | Non-adequate - Lower quality (<3) |
| Clarkson, 2006 | 1 | Non-adequate - Lower quality (<3) |
| Counsell, 2007 | 3 | Adequate -Higher quality (≥3) |
| Di Pollina, 2017 | 3 | Adequate -Higher quality (≥3) |
| Ekdahl, 2016 | 3 | Adequate -Higher quality (≥3) |
| Engelhardt, 1996 | 1 | Non-adequate - Lower quality (<3) |
| Fairhall, 2015 | 2 | Non-adequate - Lower quality (<3) |
| Fristedt, 2019 | 3 | Adequate -Higher quality (≥3) |
| Hoogendijk, 2016 | 3 | Adequate -Higher quality (≥3) |
| Imhof, 2012 | 3 | Adequate -Higher quality (≥3) |
| Li, 2010 | 1 | Non-adequate - Lower quality (<3) |
| Melis, 2008 | 3 | Adequate -Higher quality (≥3) |
| Metzelthin, 2013 | 2 | Non-adequate - Lower quality (<3) |
| Monteserin, 2010 | 3 | Adequate -Higher quality (≥3) |
| Montgomery, 2003 | 2 | Non-adequate - Lower quality (<3) |
| Orcel, 2024 | 3 | Adequate -Higher quality (≥3) |
| Reuben, 1999 | 2 | Non-adequate - Lower quality (<3) |
| Rockwood, 2000 | 2 | Non-adequate - Lower quality (<3) |
| Silverman, 1995 | 2 | Non-adequate - Lower quality (<3) |
| Sommers, 2000 | 2 | Non-adequate - Lower quality (<3) |
| Spoorenberg, 2018 | 3 | Adequate -Higher quality (≥3) |
| Stensvik, 2022 | 3 | Adequate -Higher quality (≥3) |
| Brazil, 2022 | 2 | Non-adequate - Lower quality (<3) |
| Lyndon, 2023 | 3 | Adequate -Higher quality (≥3) |
| Safari, 2023 | 3 | Adequate -Higher quality (≥3) |
| Federman, 2023 | 3 | Adequate -Higher quality (≥3) |
| Mangin, 2023 | 3 | Adequate -Higher quality (≥3) |
| De Luca, 2021 | 3 | Adequate -Higher quality (≥3) |
| Mueller, 2021 | 3 | Adequate -Higher quality (≥3) |
| Romskaug, 2019 | 3 | Adequate -Higher quality (≥3) |

In line with commonly adopted thresholds in the literature, scores of ≥3 were considered indicative of higher methodological quality, whereas scores of <3 were considered indicative of lower methodological quality (Lundh et al., 2018, De Cassai et al., 2023).

Lundh, A., Gøtzsche, P.C. Recommendations by Cochrane Review Groups for assessment of the risk of bias in studies. BMC Med Res Methodol 8, 22 (2008). <https://doi.org/10.1186/1471-2288-8-22>

De Cassai A, Boscolo A, Zarantonello F, Pettenuzzo T, Sella N, Geraldini F, Munari M, Navalesi P. Enhancing study quality assessment: an in-depth review of risk of bias tools for meta-analysis-a comprehensive guide for anesthesiologists. J Anesth Analg Crit Care. 2023 Nov 6;3(1):44. doi: 10.1186/s44158-023-00129-z. PMID: 37932825; PMCID: PMC10626791.

## Table 4. Details on CGAM instruments for each study

| **Study** | **CGAM Assessment instrument** |
| --- | --- |
| Bernabei, 1998 | Physical function: ADL (6-item), IASDL (7-item); Cognitive function and mood: SPMSQ, GDS; List of diagnoses and drug treatments and the number of home visits provided by GPs; British Columbia long term care program (modified version) |
| Boult, 2001 | Medical conditions, psychosocial status, functional ability, cognitive status, nutritional risk, use of alcohol, social network, gait and balance, environmental safety, medications, advance directives, hearing, and vision - Multidimensional Functional Assessment: the OARS Methodology, ADL, MMS, Nutrition screening instruments, CAGE questionnaire, LSNS, TUG test |
| Brazil, 2022 | No details |
| Clarkson, 2006 | MMSE; Depression: GDS (15-item version); Activities of daily living: BI; CAPEBRS; LSNS; Need Shortfall Rating |
| Counsell, 2007 | GRACE protocol: a medical and psychosocial history, medication review, functional assessment, and review of social supports and advance directives. Special attention to orthostatic vital signs, vision, hearing, gait and balance, affect, and mental status. Reference for complete assessment: Counsell et al., (2006)* |
| De Luca, 2021 | MMSE; ADL; IADL; GDS; BANSS; BPRS; MNA; BPRS; CBI; SUS |
| Di Pollina, 2017 | Cognitive function: MMSE, clock drawing test; Mood: GDS; Functional status: basic and instrumental activities of daily living; Gait: TUG test, Semi Tandem Stand; Nutrition: MNA (short form); Pain: Visual Analogue Scale; Medication review and adherence |
| Ekdahl, 2016 | Medical assessment: Problem list including dental health; MSAS; Assessment of comorbid conditions and disease severity, Medication review; BMI; Physical and neurological examination; Assessment of functioning: BI; Grip strength; Walking speed; Psychological and cognitive assessment: GDS; MMSE; Social assessment: Questions by a social worker and nurse: social support, transportation, family and other caregivers; Overall assessments: Degree of frailty: CHSA; EQ-5D; Feeling of security (patient); Feeling of security and quality of life (one relative). Assessment reported in Lind-Mazya et al., (2013)^§^. |
| Engelhardt, 1996 | Medical Outcomes Study Short-Form Health Survey; FIM; CSI; GDS, BSI; PGCMSR; LSNS; SSS, QAR; COC; ll-item PPI; SSQ; FBQ; PSQ |
| Fairhall, 2015 | Demographic and health information; Cognitive function: MMSE; Number of previous falls and fractures. Assessment reported in Fairhall et al., (2008)^ |
| Federman, 2023 | Medical history; Physical exam; Symptoms; Activities of daily living; Fall risk; MOLST |
| Fristedt, 2019 | CGAM instruments are not fully reported (reference for assessment: Ellis et al., 2015°). |
| Hoogendijk, 2016 | interRAI Community Health Assessment (CHA, version 9.1) |
| Imhof, 2012 | Demographic variables; Living situation; Family network; Health status (mobility and falls, pain, hearing ability, sleep pattern, bladder control, substance use, cognition, and use of medications and aides for mobility; Vision: Amsler-Gitter Test; Gait, balance, and strength: TUG test, tandem stand, timed five-chair-rise test; Nutrition: MNA; Depression: GDS |
| Li, 2010 | Standardized questionnaires as assessment instruments to collect information on geriatric syndromes (falls, incontinence, polypharmacy, sleep disturbance, and pain conditions); MMSE; Short-form GDS; Nutrition: MNA; Visual acuity test; TUG test; Orthostatic hypotension screening; FRT |
| Lyndon, 2023 | No details |
| Mangin, 2023 | TAP-Report (reference for assessment: Mangin et al., 2020^ç^). Demographic information; Quality of life: EQ 5D-5L; Physical activity: IPAQ (short); RAPA; Enablement: PEI; Treatment burden: MTBQ Disease burden: DBMA; Daily life activities; Goals; Social life Friendship Scale/Custom; General health Edmonton Frail Scale; Nutrition Screen II (8-item); Mobility; Sleep 15-D (sleep item); Personal health record; Social context; Memory; Advance care planning; Oral health: Recommended Oral Health Screening Questions; Smoking and alcohol Custom; Health TAPESTRY experience; Community program and service use Community Programs and Services (adapted) |
| Melis, 2008 | MMSE; GARS-3; MOS |
| Metzelthin, 2013 | Multidimensional assessment of existing problems in performing daily activities and risk factors for developing disability. PoC intervention protocol offers recommendations and guidelines for execution of treatment plan. For example, a flexible toolbox of interventions is available focusing on five topics: meaningful activities, adapting environment, activities or skills, social network and social activities, daily physical activity, stimulating health |
| Monteserin, 2010 | Sociodemographic data; Perceived health status (one question); Sensory evaluation (sight and hearing); Falls (n. of falls in the last 6 mo); Urinary incontinence (n. of events in the last 6 mo); Medications; Comorbidity: Charlson index; Functional status: BI; Instrumental activities of daily living: Lawton index; Neuropsychological evaluation: 5-Yesavage Depression Scale; Cognitive status: Short Portable Mental Status Questionnaire; Nutrition: MNA Short Form; Social support evaluation: Gijon Social Scale |
| Montgomery, 2003 | History and a functional, social, and environmental assessment; Cognitive function: MMSE; Physical functioning and self-maintenance: Home Care Rating System (a modified version of OARS ADL and IADL scales); TUG; “Functional Reach” balance test; Social support: self-reported data; Caregiver burden: Zarit Burden Scale; Family/satisfaction with Home Care services: Caregiver Satisfaction Scale |
| Mueller, 2021 | Active Geriatric Evaluation instrument; Functionality: 4 questions; Urinary incontinence: 4 questions; Mood disorder: PHQ-2; Cognitive impairment: Mini-Cog; Visual impairment: Near vision pocket card; Hearing impairment: Whisper test; Gait and balance: history of falls during past year; gait observation; Osteoporosis: history of osteoporotic fracture, height loss since age 25, Occiput–wall and rib–pelvis distance; Undernutrition: Weight loss > 5% past month or 10% past 6 months |
| Orcel, 2024 | Instruments available upon request |
| Reuben, 1999 | Instruments available upon request |
| Rockwood, 2000 | Mental status, emotional health, communication, mobility, balance, bowels, bladders, nutrition, daily activities, social situation/support, medications, environment; Cognitive function: MMSE; Physical function: BI, PSMS, IADL; Quality of life: SQLI (modified with adding/modifying domains of cognition, environment, activity and daily living); Use of formal health and social care services; Self-rated health question |
| Romskaug, 2019 | Medical history; Cognitive function: dementia, IQCODE, CDR, relatives, impression of patient, NPS; Depression/anxiety: ICD-10 criteria; Nutrition: weight loss, reduced appetite, nausea, dyspepsia, BMI, MNA-SF; Pain: previous/current problem, cause, analgesia; Breathing: dyspnea; Hydration: dehydration; overhydration/edema; Natural functions: urinary incontinence; voiding problems; diarrhea/constipation; Mobility: gait problems; dizziness; walking aids; history of falling; Sleep: any problems; Medications; Clinical examination; Supplementary tests: blood pressure (including orthostatic), pulse rate, respiratory rate; ECG; relevant blood analyses; serum concentration of relevant drugs; pharmacogenetic tests |
| Safari, 2023 | CGAM details available at: <https://bmcgeriatr.biomedcentral.com/articles/10.1186/s12877-023-04218-0#Sec25> (examples are Physical health, Medication, Bone health and falls, Function, social, environment, Mobility and balance, Care and support plan) |
| Silverman, 1995 | A comprehensive evaluation for medical, psychological, and social health problems. The assessment process included: Participant interviews; Demographic information; Functional status: ADL portion of the OARS Multi- dimensional Functional Assessment, BI; Cognitive status: MMSE, CDR3; Emotional status: clinical depression and generalized anxiety sections of the DIS; Self- perceived health status: single item addressing change in health since the last interview; Urinary and bowel incontinence: a direct question concerning frequency of incontinence |
| Sommers, 2000 | No details for CGAM assessment. The authors reported: The nurse or the social worker visited the patient at home, listened to health concerns, took vital signs and health histories, and completed a patient functional assessment and a home safety check) |
| Spoorenberg, 2018 | No details |
| Stensvik, 2022 | PSMS; CSDD; CDR scale; NPI-Q, 12-ITEMS; QUALID; The Brief Agitation Rating Scale, a subscale of CMAI; The 24-h registration of behavior form |
| GP: general practitioner; SPMSQ: Short Portable Mental Status Questionnaire; GDS: Geriatric Depression Scale; MMS: Mini-mental state; LSNS: Lubben Social Network Scale; MMSE: Standardized Mini-Mental State Examination; BI: Barthel Index; CAPEBRS: Clifton Assessment Procedures for the Elderly Behaviour Rating Scale; ADL: Activities of Daily Living; IADL: Instrumental Activities of Daily Living; BANSS: Bedford Alzheimer Nursing Severity Scale; BPRS: Brief Psychiatric Rating Scale; MNA: Mini Nutritional Assessment; BPRS: Brief Psychiatric Rating Scale; CBI: Caregiver Burden Inventory; SUS: Usability System Scale; MNA Mini-Nutritional Assessment; CHSA: Canadian Study of Health Survey; FIM: Functional Independence Measure; CSI: Computerized Severity Index; BSI: Brief Symptom Inventory; PGCMSR: Philadelphia Geriatric Center Morale Scale-Revised; SSS: Satisfaction with Support Scale; QAR: Quality Assurance Review instrument; COC: Continuity of Care Index; PPI: Pressing Problem Index; SSQ: Support Services Questionnaire; FBQ: Financial Benefits Questionnaire; PSQ: Patient Satisfaction Questionnaire; MOLST: Medical Outcomes of Life Sustaining Treatments; FRT: Functional reach test; IPAQ: International Physical Activity Questionnaire; RAPA: Rapid Assessment of Physical Activity; Enablement: PEI: Patient Enablement Instrument; MTBQ: Multimorbidity Treatment Burden Questionnaire; Disease DBMA: Burden Morbidity Assessment; GARS-3: Groningen Activity Restriction Scale; MOS: Medical Outcomes Study; PSMS Physical Self-Maintenance Scale; SQLI: Spitzer Quality of Life Index  *Counsell SR, Callahan CM, Buttar AB, Clark DO, Frank KI. Geriatric Resources for Assessment and Care of Elders (GRACE): a new model of primary care for low-income seniors. J Am Geriatr Soc. 2006 Jul;54(7):1136-41;  § Lind-Mazya A, Eckerblad J, Jaarsma T, et al. The Ambulatory Geriatric Asses- smentda Frailty Intervention Trial (AGe-FIT): A randomised controlled trial aimed to prevent hospital readmissions and functional deterioration in high risk older adults: A study protocol. Eur Geriatr Med 2013;4:242e247;  ^ Fairhall N, Aggar C, Kurrle SE, Sherrington C, Lord S, Lockwood K, Monaghan N, Cameron ID. Frailty Intervention Trial (FIT). BMC Geriatr. 2008 Oct 13;8:27; ° Ellis G, Whitehead MA, Robinson D, O'Neill D, Langhorne P. Comprehensive geriatric assessment for older adults admitted to hospital: meta-analysis of randomised controlled trials. BMJ. 2011 Oct 27;343:d6553;  ç Mangin D, Lamarche L, Oliver D, Bomze S, Borhan S, Browne T, Carr T, Datta J, Dolovich L, Howard M, Marentette-Brown S, Risdon C, Talat S, Tarride JE, Thabane L, Valaitis R, Price D. Health TAPESTRY Ontario: protocol for a randomized controlled trial to test reproducibility and implementation. Trials. 2020 Aug 14;21(1):714 | |
